# Supplementary material for: PROTACs in the Management of Prostate Cancer
Source: Molecules. 2023 Apr 25;28(9):3698. doi: 10.3390/molecules28093698 (PMC10179857; doi:10.3390/molecules28093698)
Supplement: Supplementary file 1 [file molecules-28-03698-s001.zip › molecules-2300063-supplementary.pdf]

PROTACs in the management of Prostate Cancer

Supplementary Information

Supplementary Table S1. PROTACs that target different biological proteins involved in the progression of prostate cancer.

S.No	***PROTAC***	***E3 ligase ligand***	***Biological Target***	***Year***
1	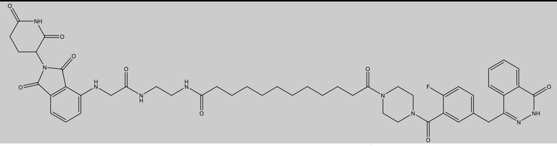	Thalidomide	PARP1 [1]	2020
2	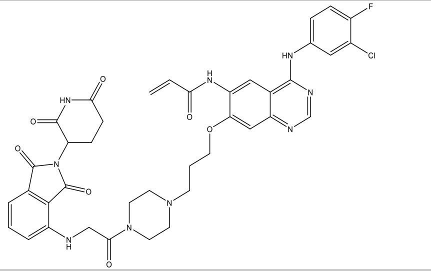	Pomalidomide	EGFR [2]	2021
3	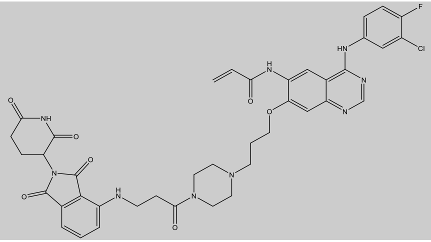	Pomalidomide	EGFR [2]	2021

4	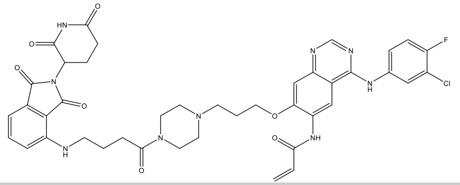	Pomalidomide	EGFR [2]	2021
5	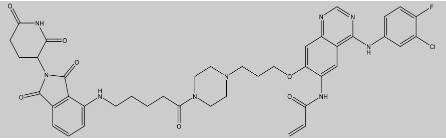	Pomalidomide	EGFR [2]	2021
6	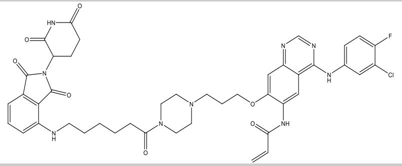	Pomalidomide	EGFR [2]	2021
7	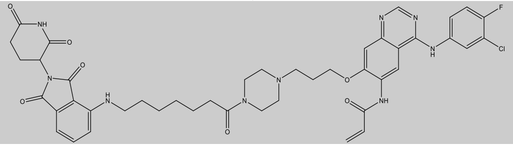	Pomalidomide	EGFR [2]	2021
8	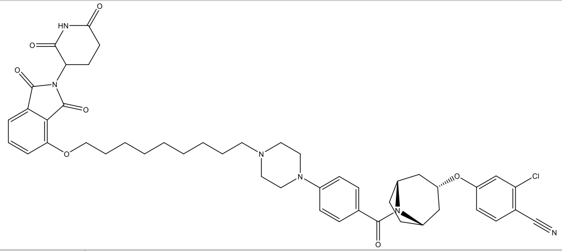	CRBN	Androgen receptor [3]	2021
9	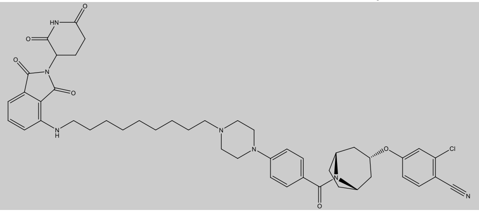	CRBN	Androgen receptor [3]	2021

10	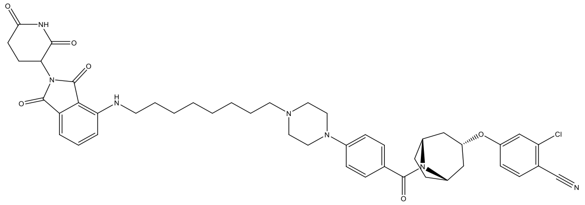	CRBN	Androgen receptor [3]	2021
11	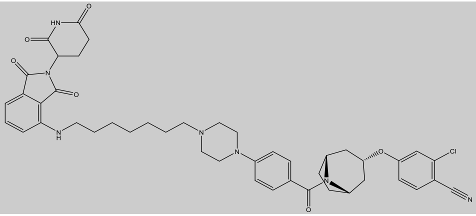	CRBN	Androgen receptor [3]	2021
12	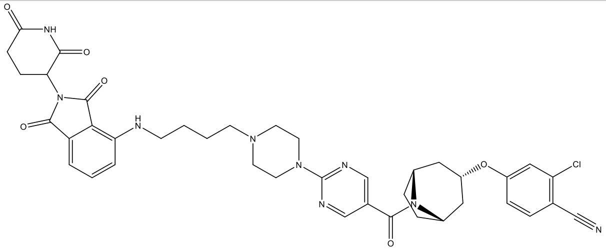	CRBN	Androgen receptor [4]	2021
13	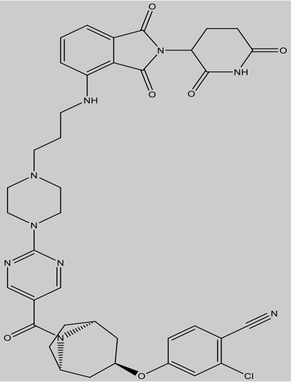	CRBN	Androgen receptor [5]	2021

14	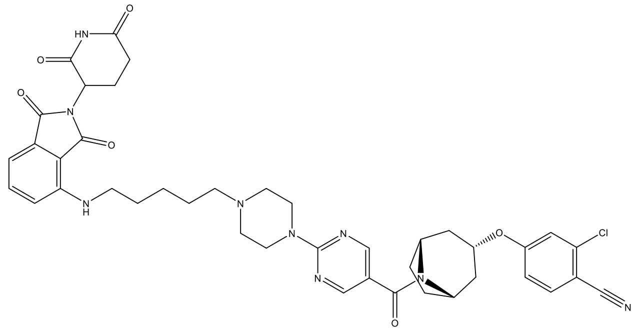	CRBN	Androgen receptor [6]	2021
15	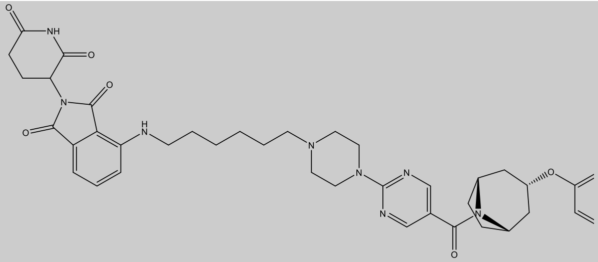	CRBN	Androgen receptor [7]	2021
16	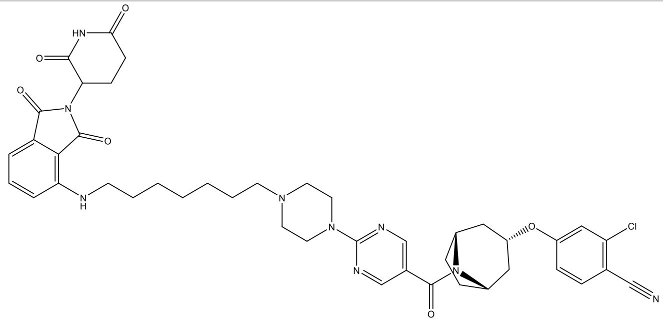	CRBN	Androgen receptor [8]	2021
17	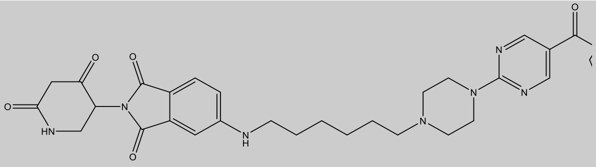	CRBN	Androgen receptor [9]	2021

18	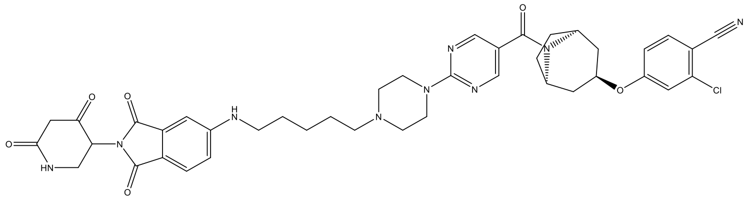	CRBN	Androgen receptor [10]	2021
19	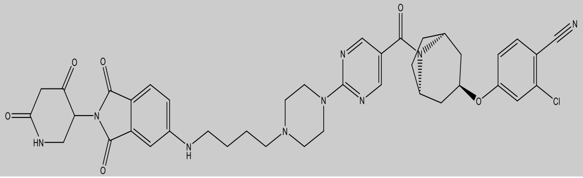	CRBN	Androgen receptor [11]	2021
20	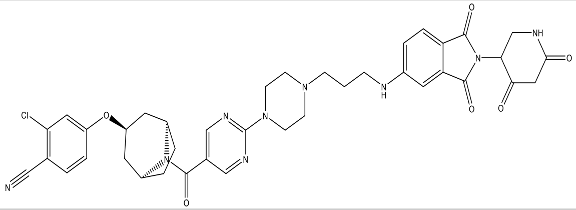	CRBN	Androgen receptor [12]	2021
21	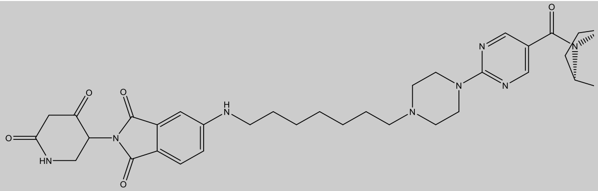	CRBN	Androgen receptor [13]	2021
22	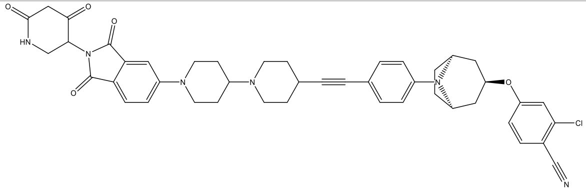	CRBN	Androgen receptor [14]	2021

23	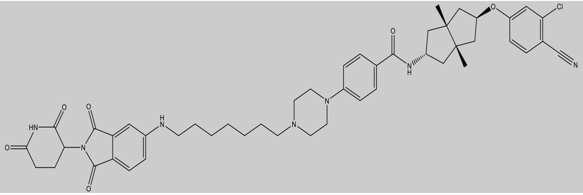	CRBN	Androgen receptor [15]	2021
24	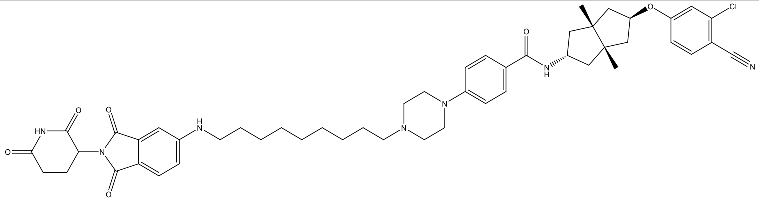	CRBN	Androgen receptor [16]	2021
25	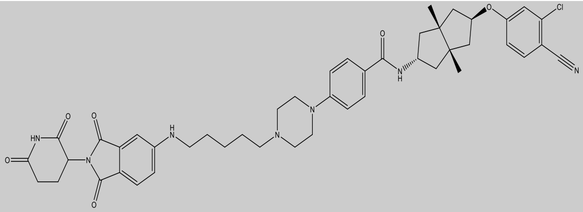	CRBN	Androgen receptor [17]	2021
26	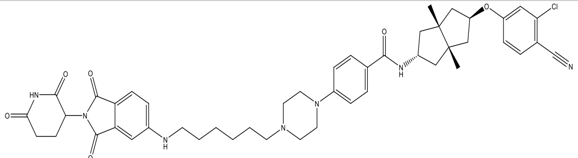	CRBN	Androgen receptor [18]	2021
27	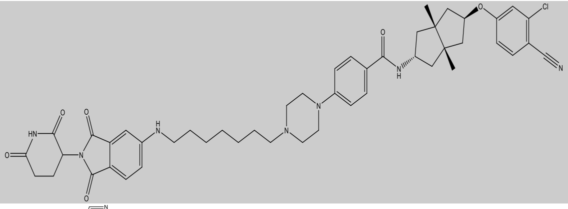	CRBN	Androgen receptor [18]	2021
28	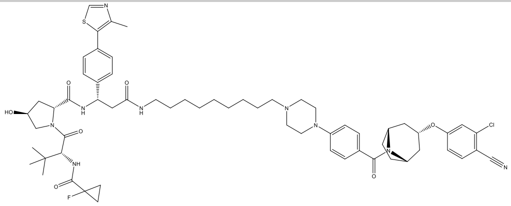	VHL-1	Androgen receptor [18]	2021

29	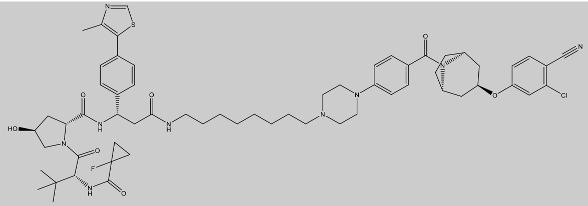	VHL-1	Androgen receptor [19]	2021
30	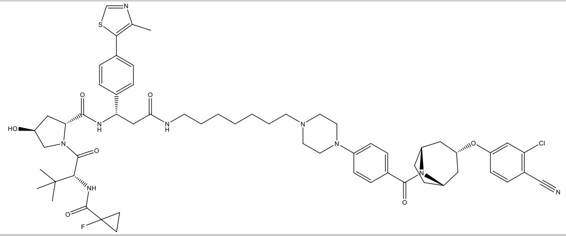	VHL-1	Androgen receptor [20]	2021
31	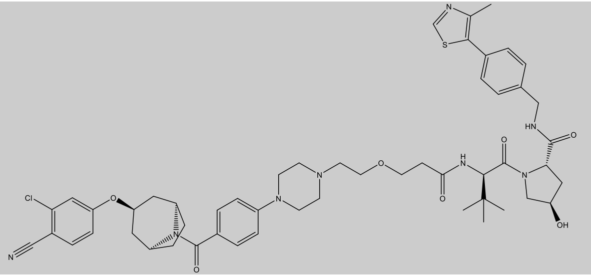	VHL-1	Androgen receptor [21]	2021
32	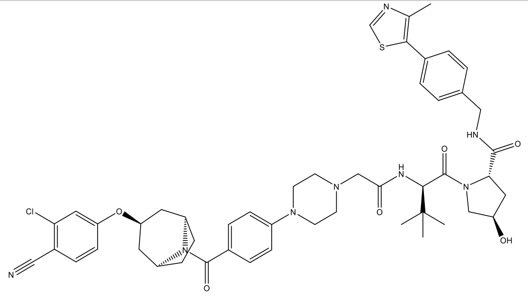	VHL-1	Androgen receptor [2]	2021

33	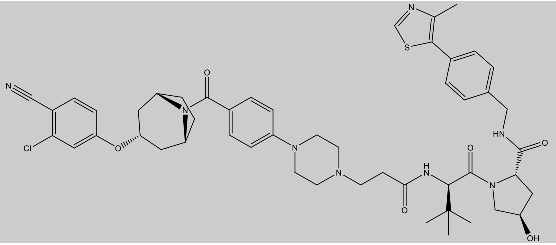	VHL-1	Androgen receptor [22]	2021
34	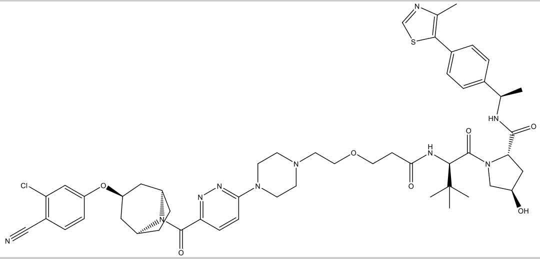	VHL-1	Androgen receptor [23]	2021
35	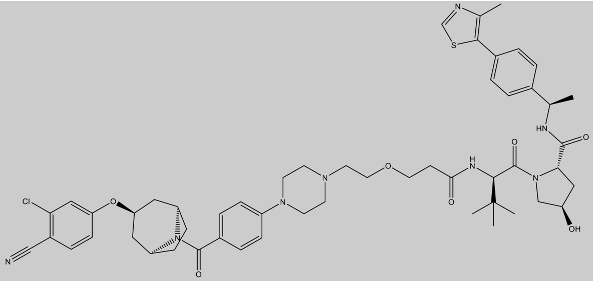	VHL-1	Androgen receptor [24]	2021
36	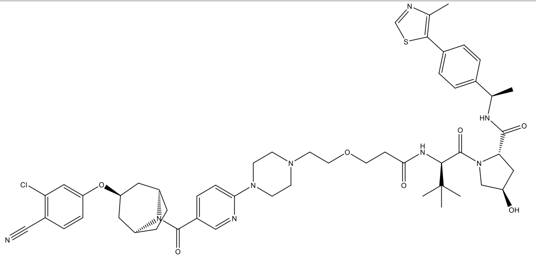	VHL-1	Androgen receptor [25]	2021

37	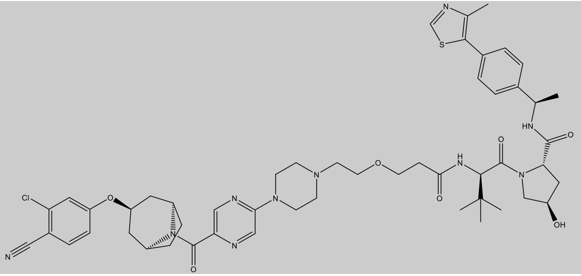	VHL-1	Androgen receptor [26]	2021
38	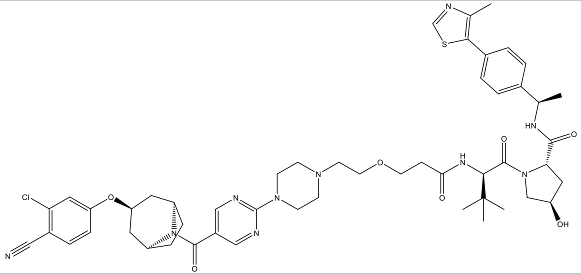	VHL-1	Androgen receptor [27]	2021
39	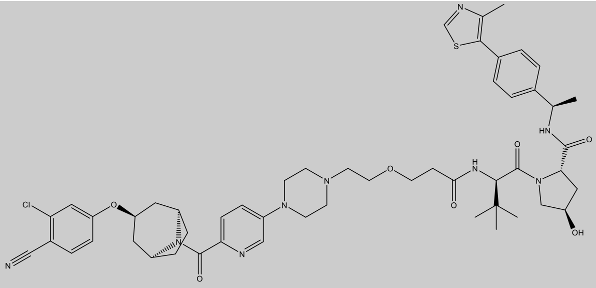	VHL-1	Androgen receptor [28]	2021
40	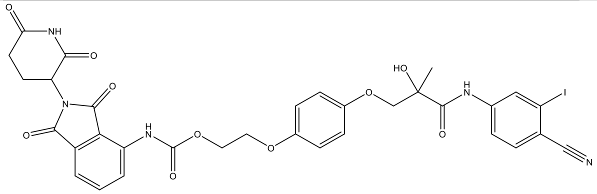	Pomalidomide	Androgen receptor [29]	2021

41	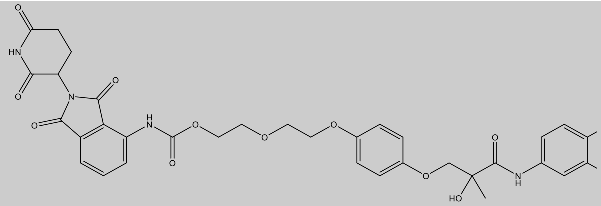	Pomalidomide	Androgen receptor [30]	2021
42	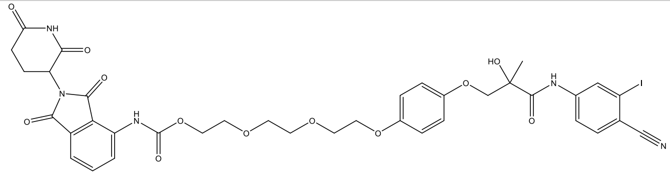	Pomalidomide	Androgen receptor [31]	2021
43	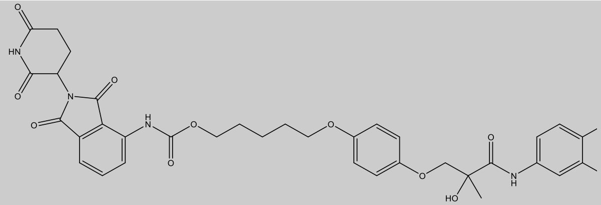	Pomalidomide	Androgen receptor [32]	2021
44	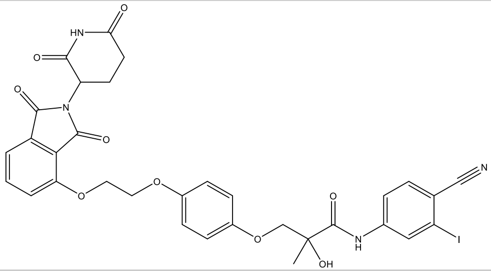	4-Hydroxy Thalidomide	Androgen receptor [33]	2021
45	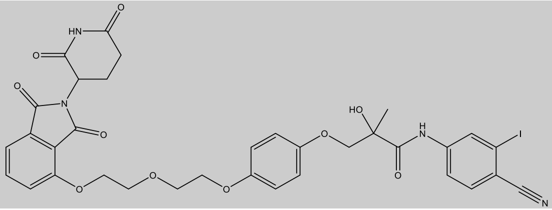	4-Hydroxy Thalidomide	Androgen receptor [34]	2021

46	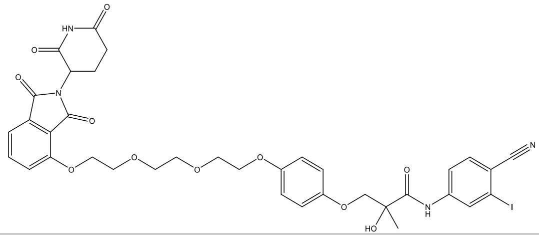	4-Hydroxy Thalidomide	Androgen receptor [35]	2021
47	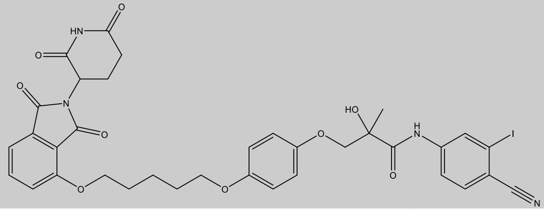	4-Hydroxy Thalidomide	Androgen receptor [36]	2021
48	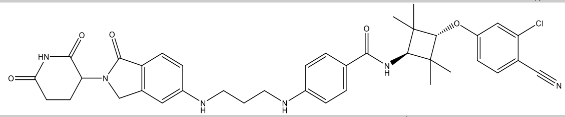	Thalidomide	Androgen receptor [37]	2021
49	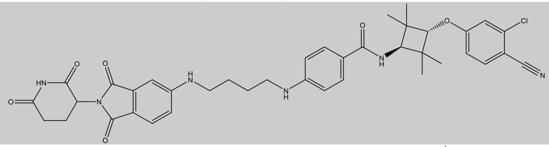	Thalidomide	Androgen receptor [38]	2021
50	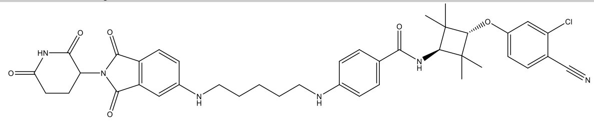	Thalidomide	Androgen receptor [39]	2021
51	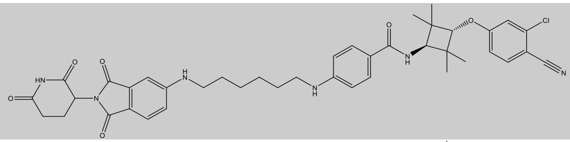	Thalidomide	Androgen receptor [39]	2021
52	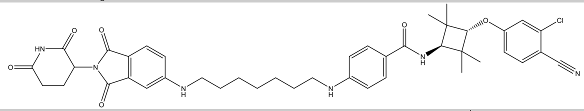	Thalidomide	Androgen receptor [40]	2021
53	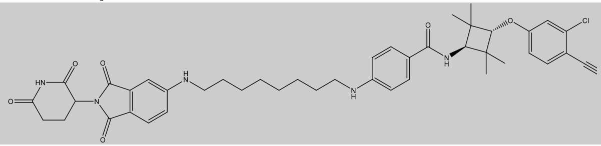	Thalidomide	Androgen receptor [41]	2021

54	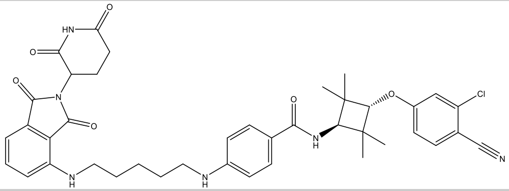	Thalidomide	Androgen receptor [42]	2021
55	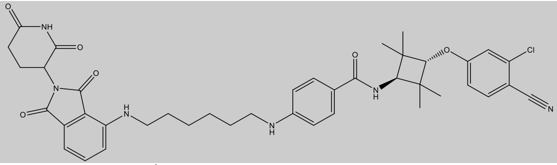	Thalidomide	Androgen receptor [43]	2021
56	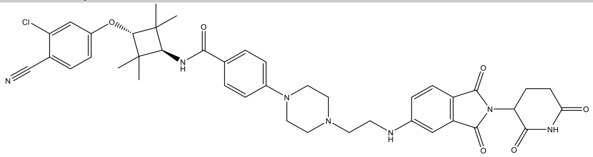	Thalidomide	Androgen receptor [44]	2021
57	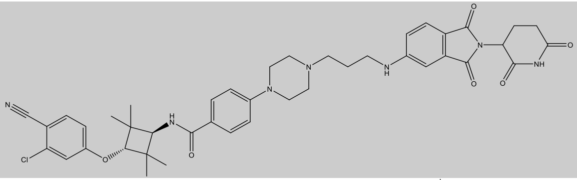	Thalidomide	Androgen receptor [44]	2021
58	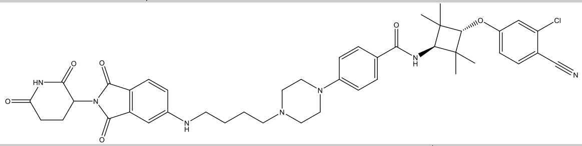	Thalidomide	Androgen receptor [45]	2021
59	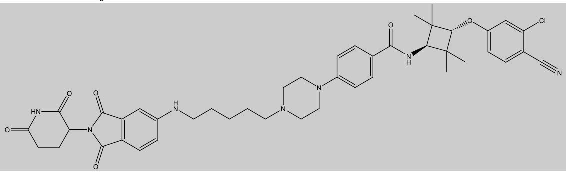	Thalidomide	Androgen receptor [45]	2021

60	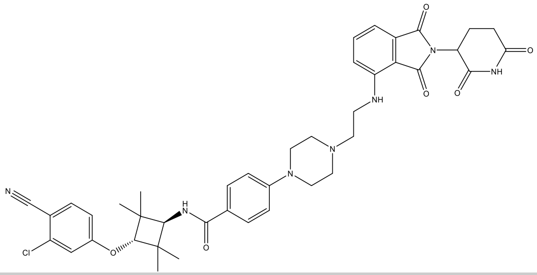	Thalidomide	Androgen receptor [45]	2021
61	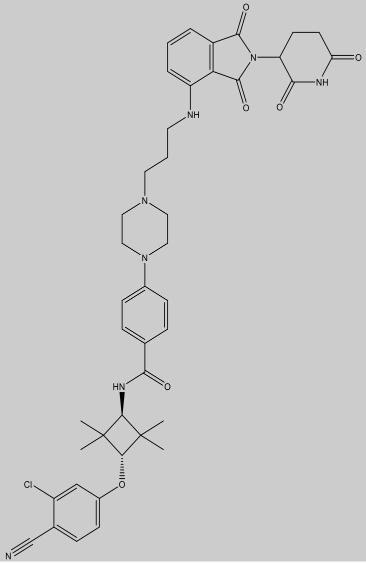	Thalidomide	Androgen receptor [46]	2021
62	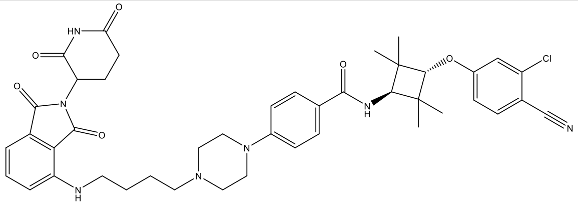	Thalidomide	Androgen receptor [47]	2021

63	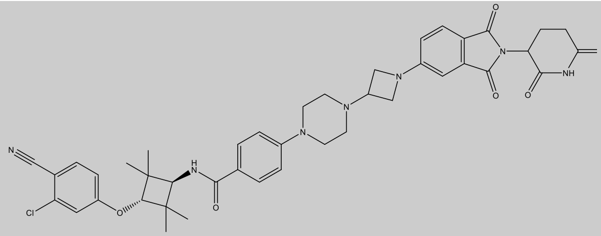	Thalidomide	Androgen receptor [47]	2021
64	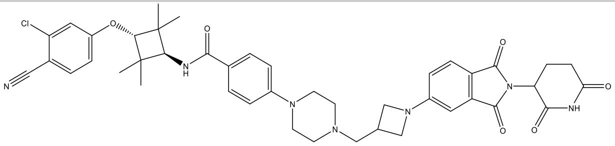	Thalidomide	Androgen receptor [47]	2021
65	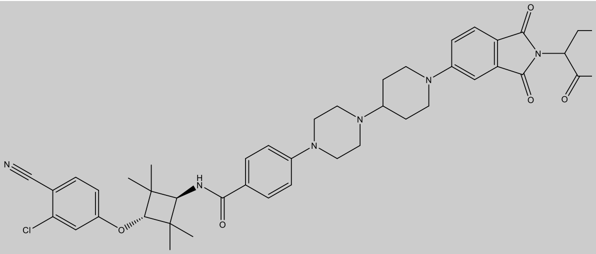	Thalidomide	Androgen receptor [47]	2021
66	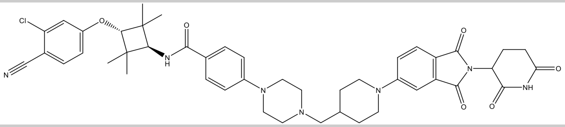	Thalidomide	Androgen receptor [48]	2021
67	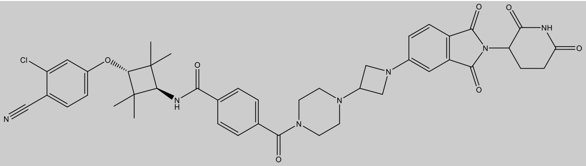	Thalidomide	Androgen receptor [49]	2021
68	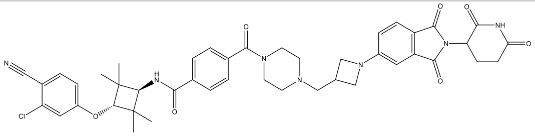	Thalidomide	Androgen receptor [49]	2021

69	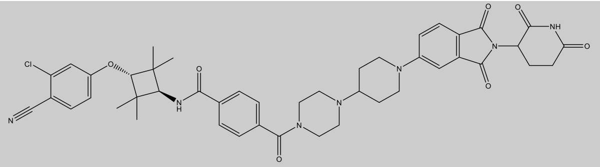	Thalidomide	Androgen receptor [49]	2021
70	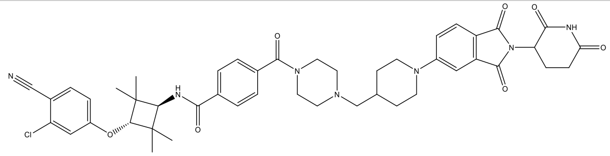	Thalidomide	Androgen receptor [50]	2021
71	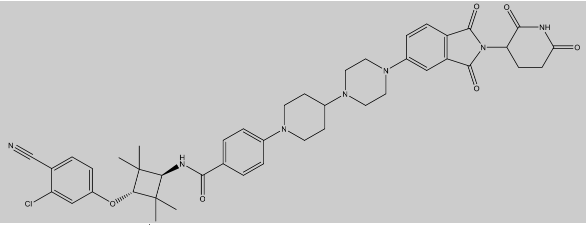	Thalidomide	Androgen receptor [51]	2021
72	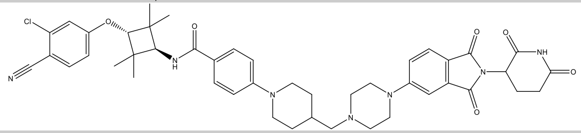	Thalidomide	Androgen receptor [52]	2021
73	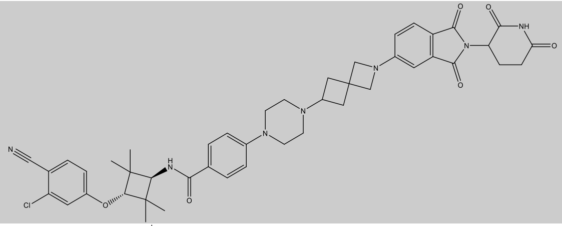	Thalidomide	Androgen receptor [52]	2021
74	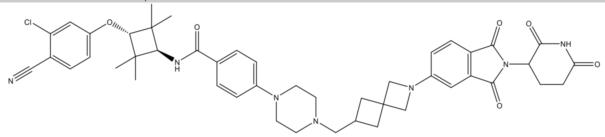	Thalidomide	Androgen receptor [52]	2021

75	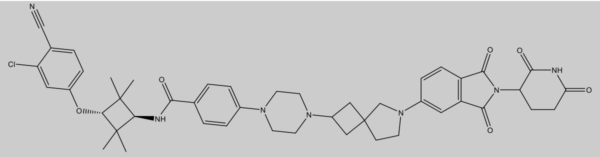	Thalidomide	Androgen receptor [52]	2021
76	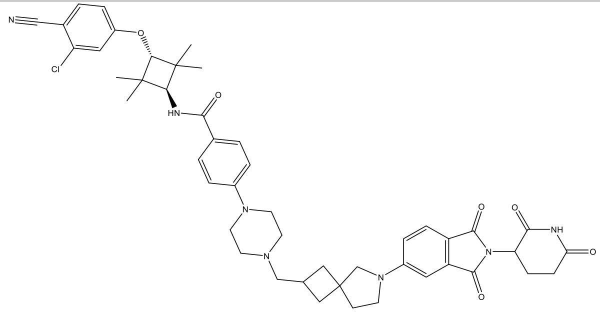	Thalidomide	Androgen receptor [52]	2021
77	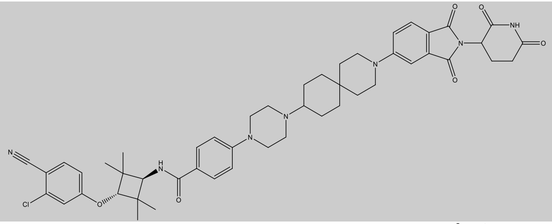	Thalidomide	Androgen receptor [53]	2021
78	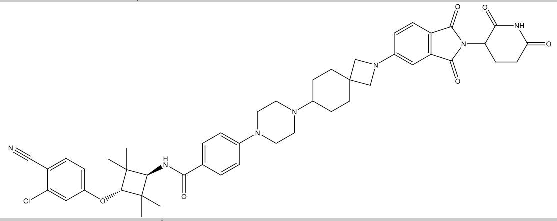	Thalidomide	Androgen receptor [53]	2021
79	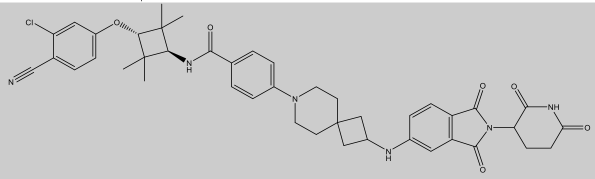	Thalidomide	Androgen receptor [53]	2021

80	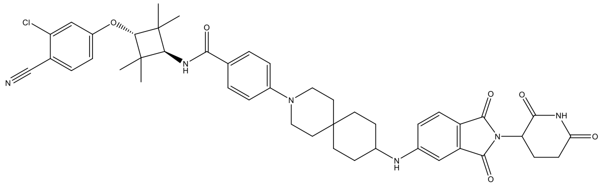	Thalidomide	Androgen receptor [54]	2021
81	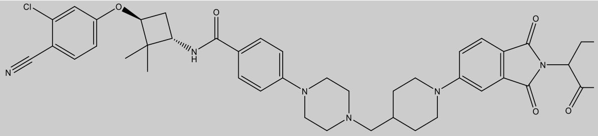	Thalidomide	Androgen receptor [54]	2021
82	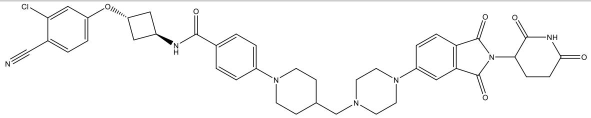	Thalidomide	Androgen receptor [54]	2021
83	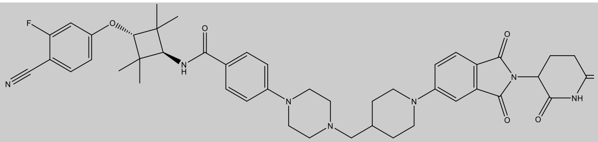	Thalidomide	Androgen receptor [55]	2021
84	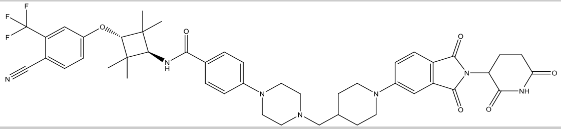	Thalidomide	Androgen receptor [56]	2021
85	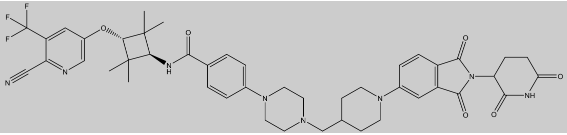	Thalidomide	Androgen receptor [56]	2021
86	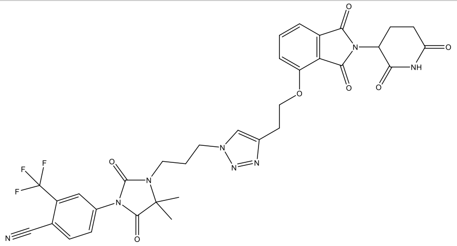	Thalidomide	Androgen receptor [57]	2021

87	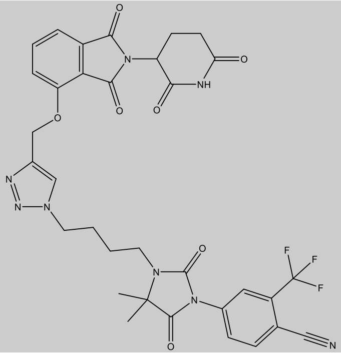	Thalidomide	Androgen receptor [58]	2021
88	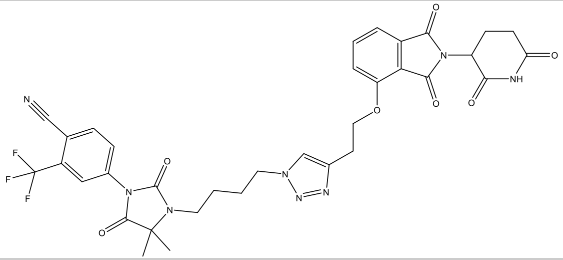	Thalidomide	Androgen receptor [58]	2021
89	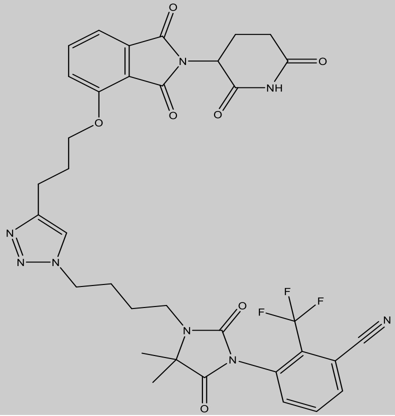	Thalidomide	Androgen receptor [58]	2021

90	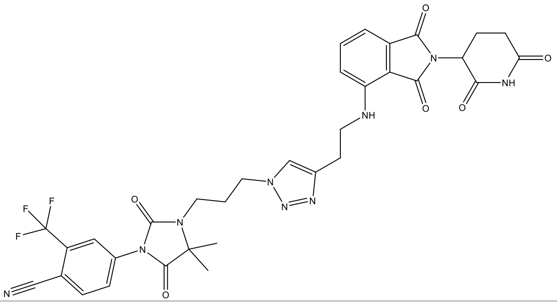	Thalidomide	Androgen receptor [58]	2021
91	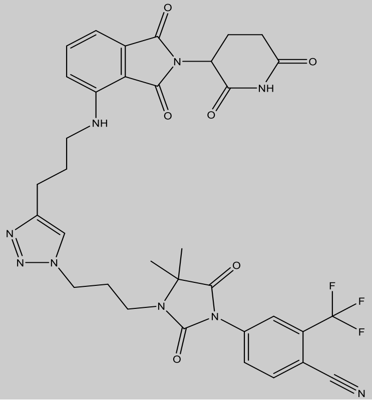	Thalidomide	Androgen receptor [58]	2021
92	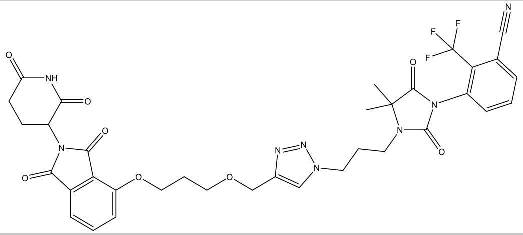	Thalidomide	Androgen receptor [58]	2021
93	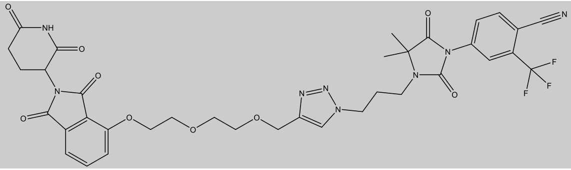	Thalidomide	Androgen receptor [58]	2021

94	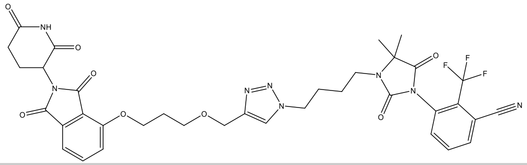	Thalidomide	Androgen receptor [58]	2021
95	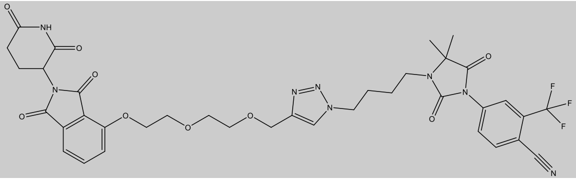	Thalidomide	Androgen receptor [58]	2021
96	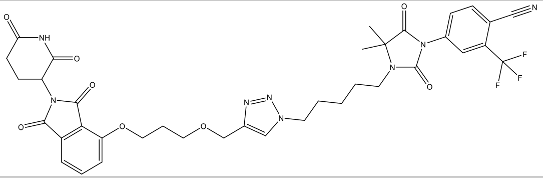	Thalidomide	Androgen receptor [59]	2021
97	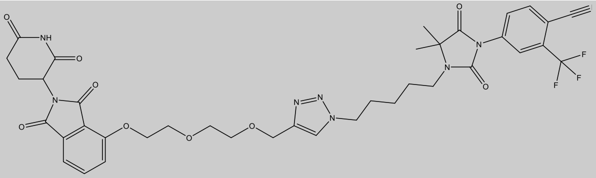	Thalidomide	Androgen receptor [60]	2021
98	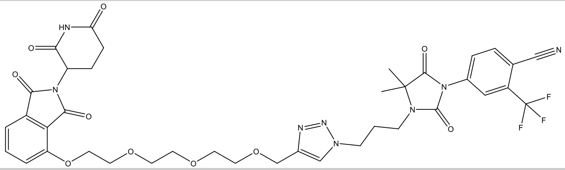	Thalidomide	Androgen receptor [61]	2021
99	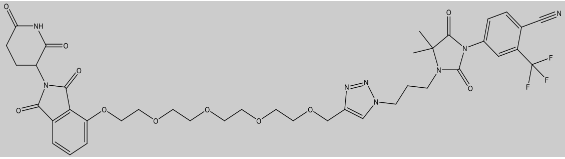	Thalidomide	Androgen receptor [62]	2021
100	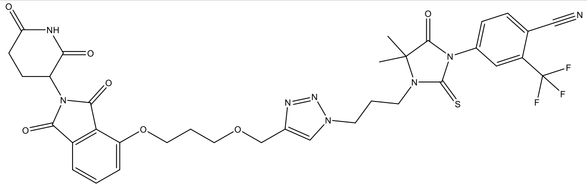	Thalidomide	Androgen receptor [63]	2021

101	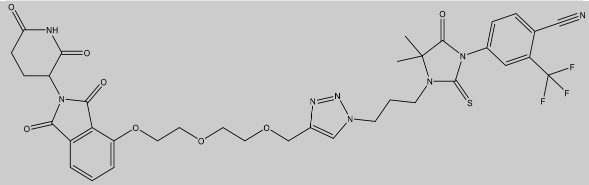	Thalidomide	Androgen receptor [64]	2021
102	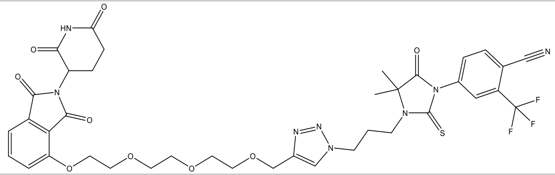	Thalidomide	Androgen receptor [65]	2021
103	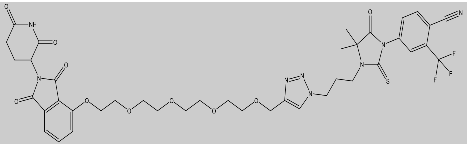	Thalidomide	Androgen receptor [66]	2021
104	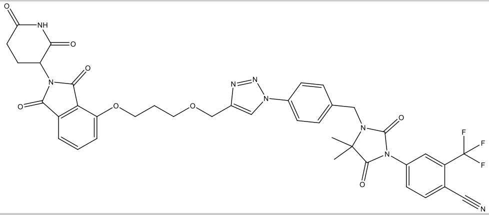	Thalidomide	Androgen receptor [67]	2021
105	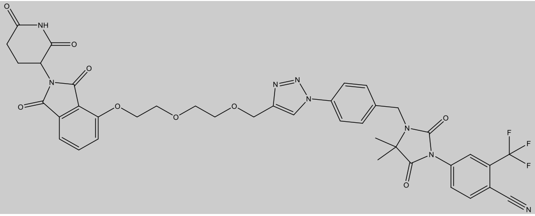	Thalidomide	Androgen receptor [68]	2021
106	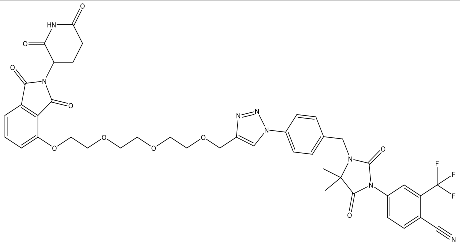	Thalidomide	Androgen receptor [69]	2021

107	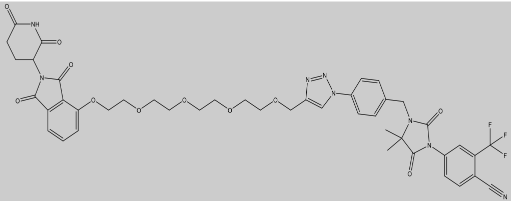	Thalidomide	Androgen receptor [70]	2021
108	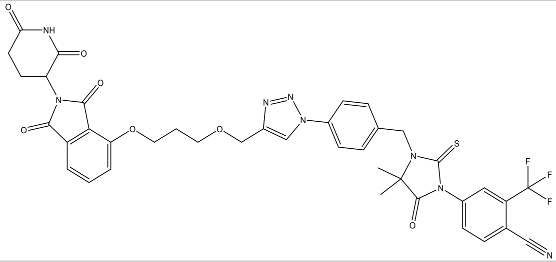	Thalidomide	Androgen receptor [71]	2021
109	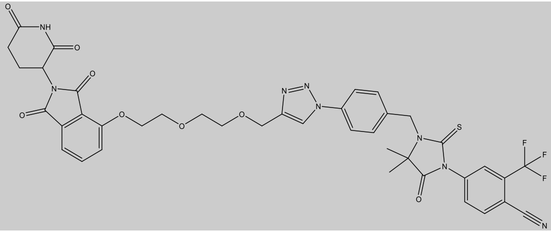	Thalidomide	Androgen receptor [72]	2021
110	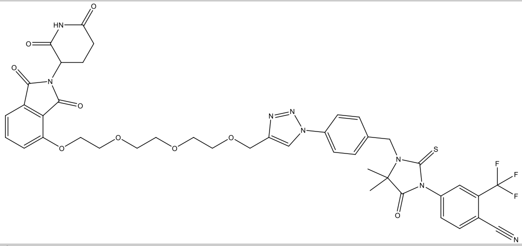	Thalidomide	Androgen receptor [73]	2021
111	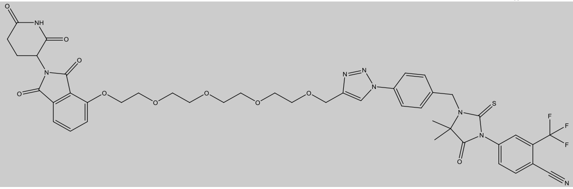	Thalidomide	Androgen receptor [74]	2021

112	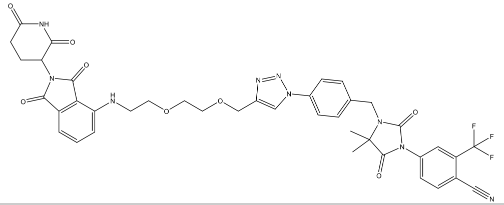	Thalidomide	Androgen receptor [75]	2021
113	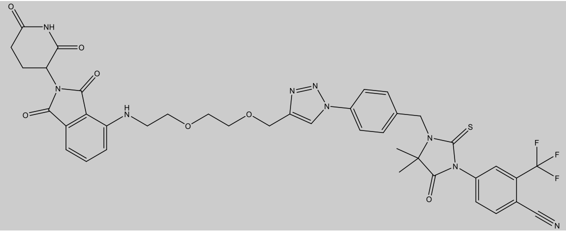	Thalidomide	Androgen receptor [76]	2021
114	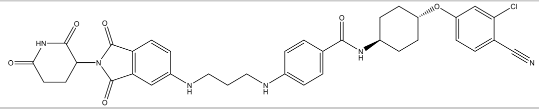	Thalidomide	Androgen receptor [77]	2021
115	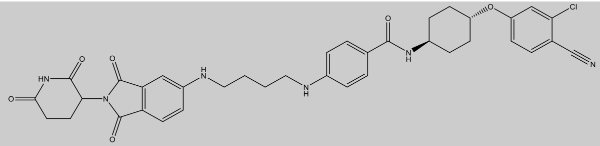	Thalidomide	Androgen receptor [48]	2021
116	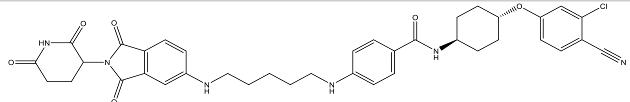	Thalidomide	Androgen receptor [49]	2021
117	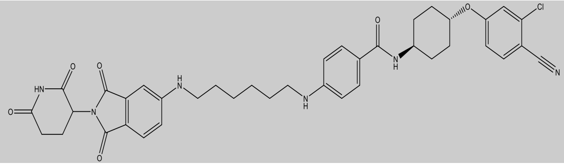	Thalidomide	Androgen receptor [49]	2021
118	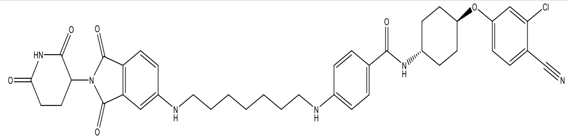	Thalidomide	Androgen receptor [50]	2021

119	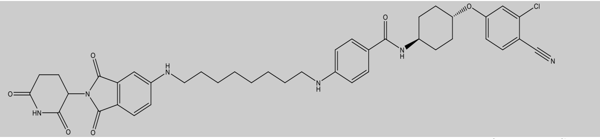	Thalidomide	Androgen receptor [51]	2021
120	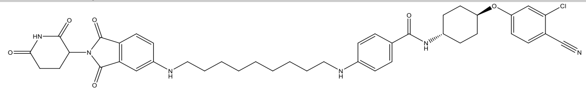	Thalidomide	Androgen receptor [52]	2021
121	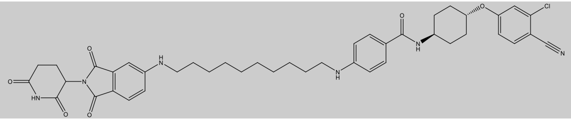	Thalidomide	Androgen receptor [52]	2021
122	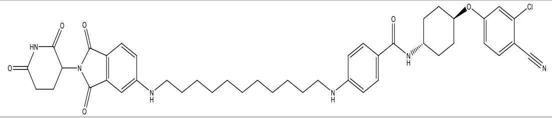	Thalidomide	Androgen receptor [52]	2021
123	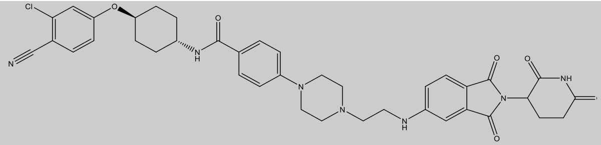	Thalidomide	Androgen receptor [52]	2021
124	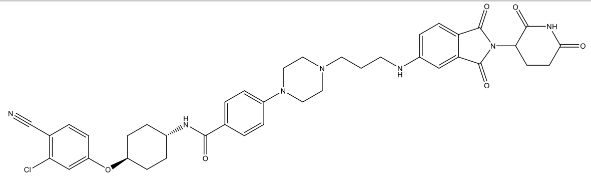	Thalidomide	Androgen receptor [52]	2021
125	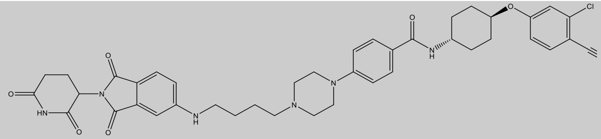	Thalidomide	Androgen receptor [53]	2021
126	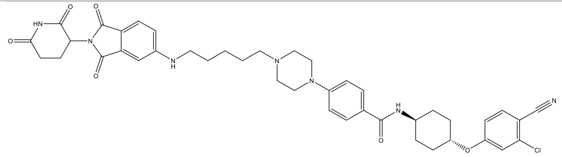	Thalidomide	Androgen receptor [53]	2021

127	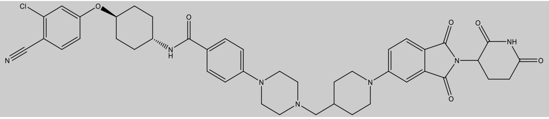	Thalidomide	Androgen receptor [53]	2021
128	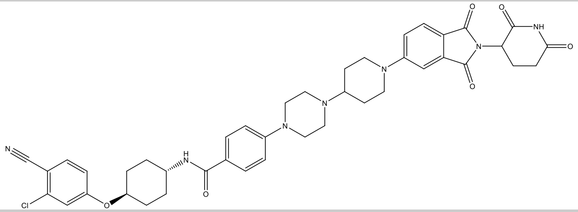	Thalidomide	Androgen receptor [54]	2021
129	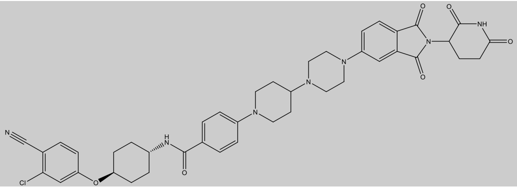	Thalidomide	Androgen receptor [54]	2021
130	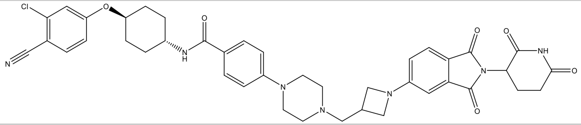	Thalidomide	Androgen receptor [54]	2021
131	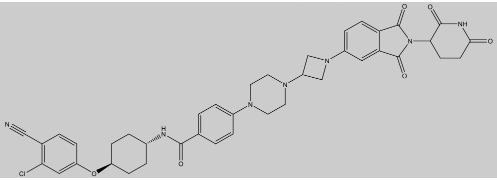	Thalidomide	Androgen receptor [54]	2021
132	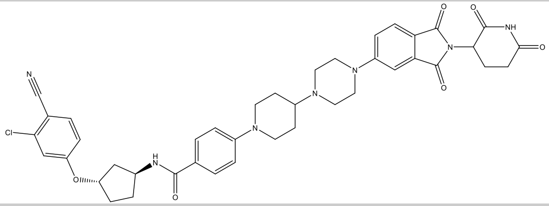	Thalidomide	Androgen receptor [55]	2021
133	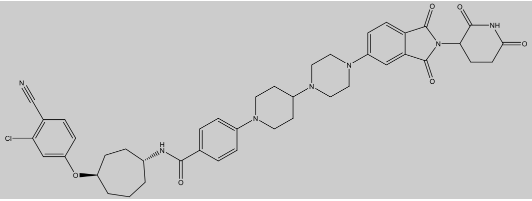	Thalidomide	Androgen receptor [56]	2021

134	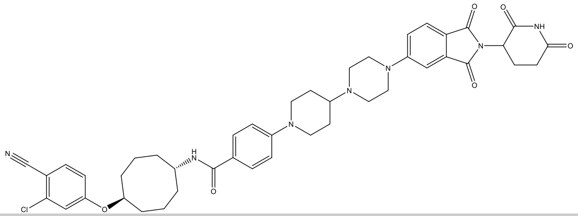	Thalidomide	Androgen receptor [56]	2021
135	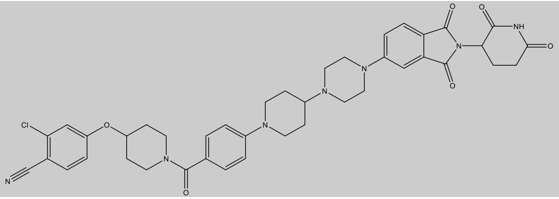	Thalidomide	Androgen receptor [57]	2021
136	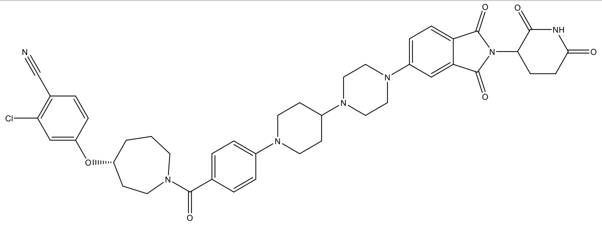	Thalidomide	Androgen receptor [57]	2021
137	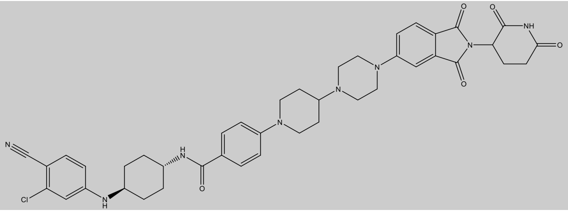	Thalidomide	Androgen receptor [57]	2021
138	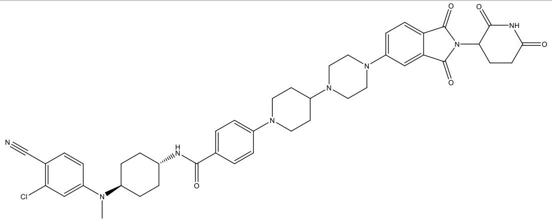	Thalidomide	Androgen receptor [57]	2021

139	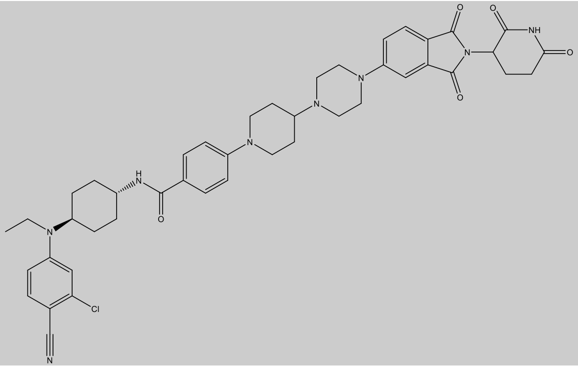	Thalidomide	Androgen receptor [57]	2021
140	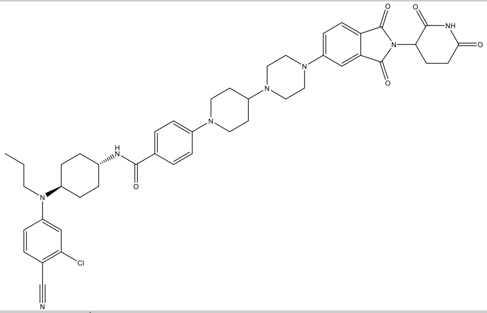	Thalidomide	Androgen receptor [57]	2021
141	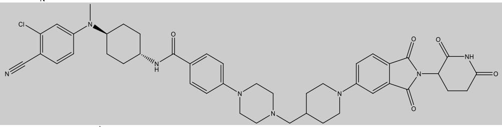	Thalidomide	Androgen receptor [78]	2021
142	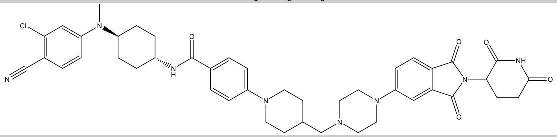	Thalidomide	Androgen receptor [79]	2021
143	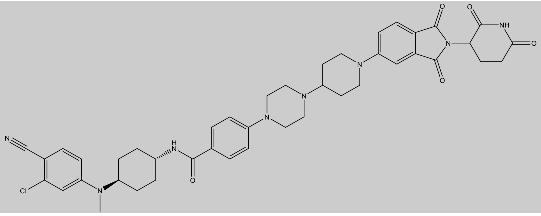	Thalidomide	Androgen receptor [79]	2021

144	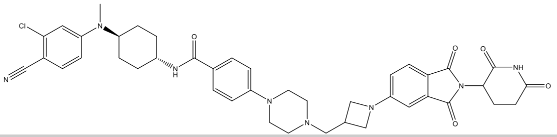	Thalidomide	Androgen receptor [79]	2021
145	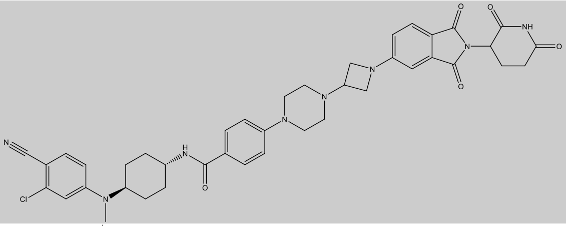	Thalidomide	Androgen receptor [79]	2021
146	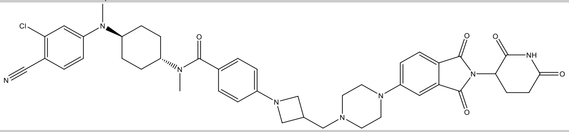	Thalidomide	Androgen receptor [79]	2021
147	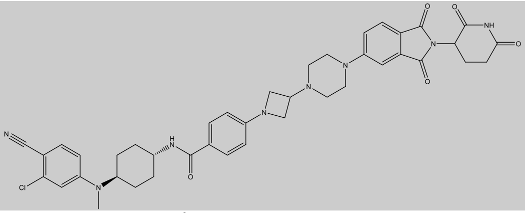	Thalidomide	Androgen receptor [80]	2021
148	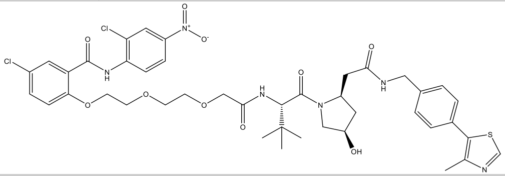	VHL	Androgen receptor [81]	2022
149	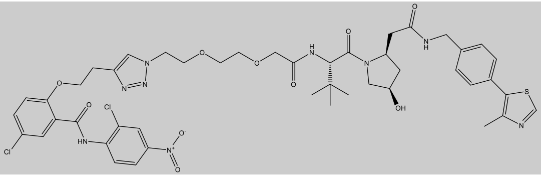	VHL	Androgen receptor [82]	2022
150	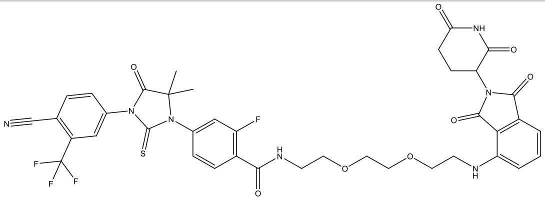	Thalidomide	Androgen receptor [83]	2022

151	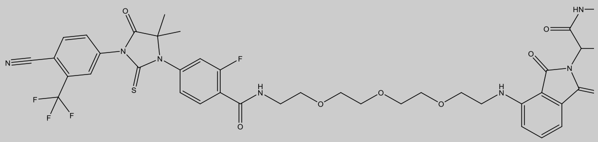	Thalidomide	Androgen receptor [84]	2022
152	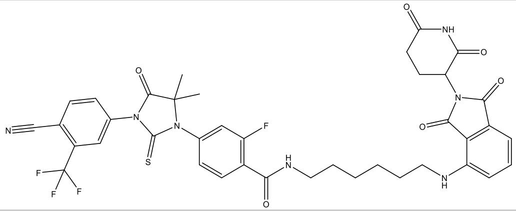	Thalidomide	Androgen receptor [85]	2022
153	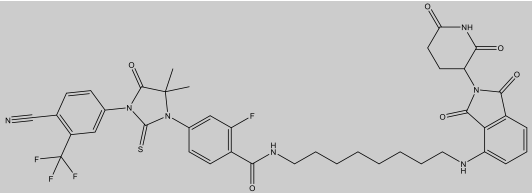	Thalidomide	Androgen receptor [86]	2022
154	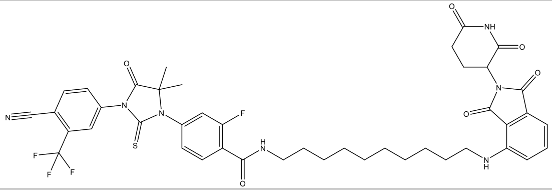	Thalidomide	Androgen receptor [87]	2022
155	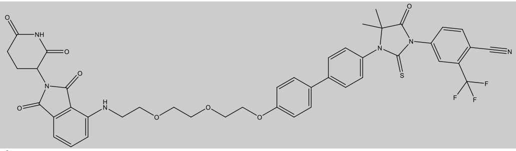	Thalidomide	Androgen receptor [87]	2022
156	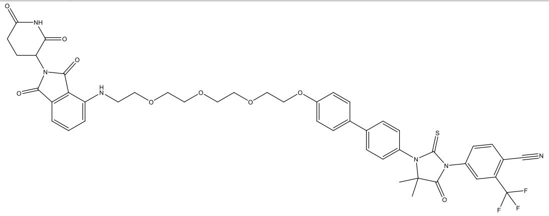	Thalidomide	Androgen receptor [87]	2022

157	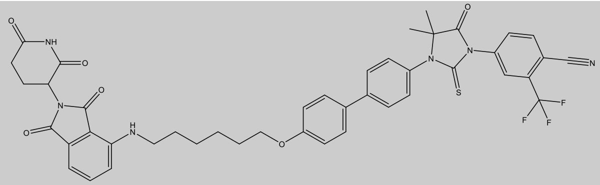	Thalidomide	Androgen receptor [87]	2022
158	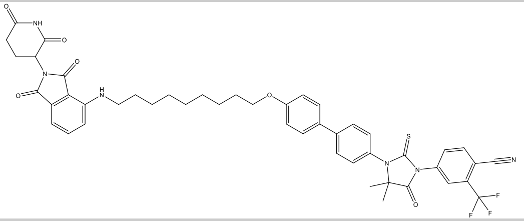	Thalidomide	Androgen receptor [87]	2022
159	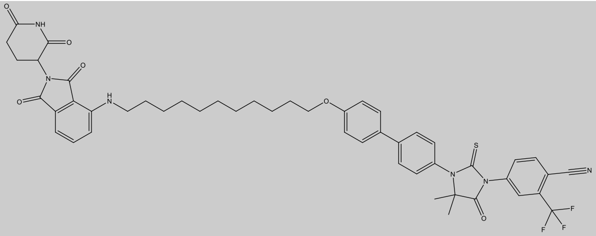	Thalidomide	Androgen receptor [87]	2022
160	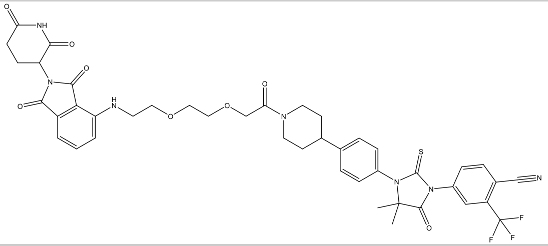	Thalidomide	Androgen receptor [88]	2022
161	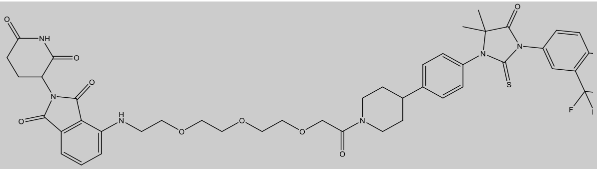	Thalidomide	Androgen receptor [88]	2022

162	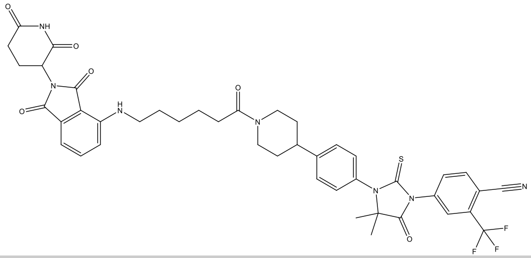	Thalidomide	Androgen receptor [88]	2022
163	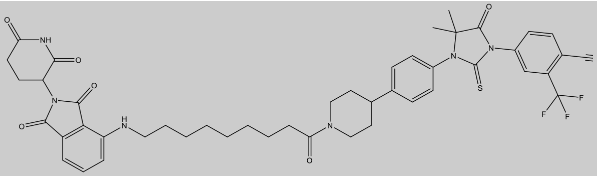	Thalidomide	Androgen receptor [88]	2022
164	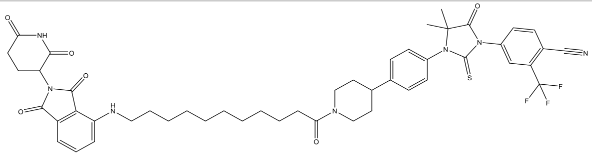	Thalidomide	Androgen receptor [88]	2022
165	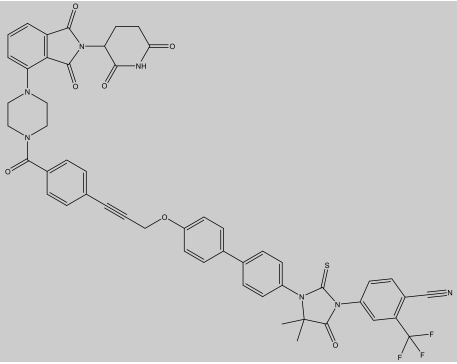	Thalidomide	Androgen receptor [89]	2022

166	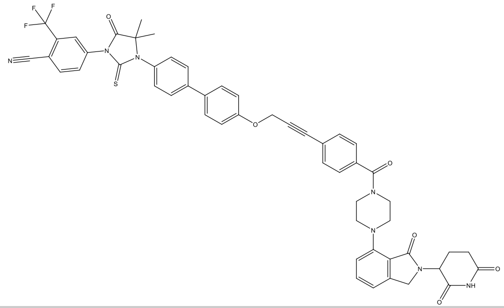	Thalidomide	Androgen receptor [89]	2022
167	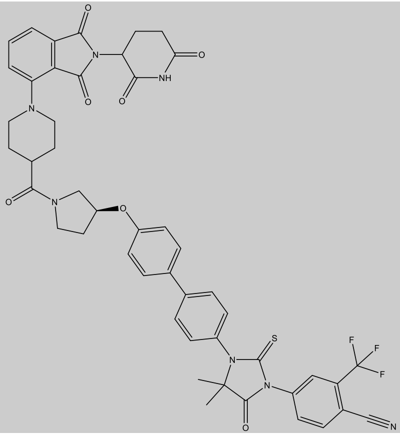	Thalidomide	Androgen receptor [89]	2022
168	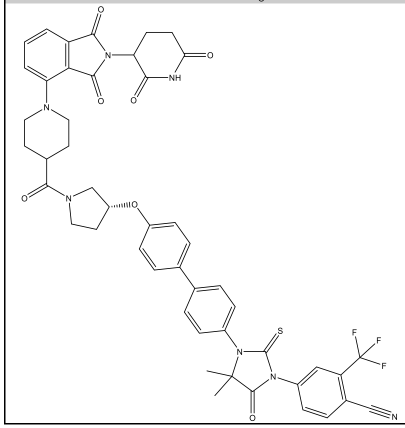	Thalidomide	Androgen receptor [89]	2022

<u>**References:**</u>

1. Cao, C.; Yang, J.; Chen, Y.; Zhou, P.; Wang, Y.; Du, W.; Zhao, L.; Chen, Y., Discovery of SK-575 as a Highly Potent and Efficacious Proteolysis-Targeting Chimera Degrader of PARP1 for Treating Cancers. *Journal of medicinal chemistry* **2020,** 63, (19), 11012-11033.
2. Qu, X.; Liu, H.; Song, X.; Sun, N.; Zhong, H.; Qiu, X.; Yang, X.; Jiang, B., Effective degradation of EGFR(L858R+T790M) mutant proteins by CRBN-based PROTACs through both proteosome and autophagy/lysosome degradation systems. *European journal of medicinal chemistry* **2021,** 218, 113328.
3. Chen, L.; Han, L.; Mao, S.; Xu, P.; Xu, X.; Zhao, R.; Wu, Z.; Zhong, K.; Yu, G.; Wang, X., Discovery of A031 as effective proteolysis targeting chimera (PROTAC) androgen receptor (AR) degrader for the treatment of prostate cancer. *European journal of medicinal chemistry* **2021,** 216, 113307.
4. Sohrabi, M.; Saeedi, M.; Larijani, B.; Mahdavi, M., Recent advances in biological activities of rhodium complexes: Their applications in drug discovery research. *European journal of medicinal chemistry* **2021,** 216, 113308.
5. Jansa, J.; Jorda, R.; Skerlova, J.; Pachl, P.; Perina, M.; Reznickova, E.; Heger, T.; Gucky, T.; Rezacova, P.; Lycka, A.; Krystof, V., Imidazo[1,2-c]pyrimidin-5(6H)-one inhibitors of CDK2: Synthesis, kinase inhibition and co-crystal structure. *European journal of medicinal chemistry* **2021,** 216, 113309.
6. Sang, Z.; Song, Q.; Cao, Z.; Deng, Y.; Tan, Z.; Zhang, L., Design, synthesis and evaluation of novel dimethylamino chalcone-O-alkylamines derivatives as potential multifunctional agents against Alzheimer's disease. *European journal of medicinal chemistry* **2021,** 216, 113310.
7. Liu, Y. Y.; Guo, Z.; Wang, J. Y.; Wang, H. M.; Da Qi, J.; Ma, J.; Piao, H. R.; Jin, C. H.; Jin, X., Synthesis and evaluation of the epithelial-to-mesenchymal inhibitory activity of indazole-derived imidazoles as dual ALK5/p38alpha MAP inhibitors. *European journal of medicinal chemistry* **2021,** 216, 113311.
8. Singh, S. S.; Mattheolabakis, G.; Gu, X.; Withers, S.; Dahal, A.; Jois, S., A grafted peptidomimetic for EGFR heterodimerization inhibition: Implications in NSCLC models. *European journal of medicinal chemistry* **2021,** 216, 113312.
9. Lu, R.; Wang, Y.; Liu, C.; Zhang, Z.; Li, B.; Meng, Z.; Jiang, C.; Hu, Q., Design, synthesis and evaluation of 3-amide-5-aryl benzoic acid derivatives as novel P2Y(14)R antagonists with potential high efficiency against acute gouty arthritis. *European journal of medicinal chemistry* **2021,** 216, 113313.
10. Zhang, Z.; Xing, X.; Guan, P.; Song, S.; You, G.; Xia, C.; Liu, T., Recent progress in agents targeting polo-like kinases: Promising therapeutic strategies. *European journal of medicinal chemistry* **2021,** 217, 113314.
11. Villard, A. L.; Aubertin, A. M.; Peyrottes, S.; Perigaud, C., An original pronucleotide strategy for the simultaneous delivery of two bioactive drugs. *European journal of medicinal chemistry* **2021,** 216, 113315.
12. Zhu, H.; Li, W.; Shuai, W.; Liu, Y.; Yang, L.; Tan, Y.; Zheng, T.; Yao, H.; Xu, J.; Zhu, Z.; Yang, D. H.; Chen, Z. S.; Xu, S., Discovery of novel N-benzylbenzamide derivatives as tubulin polymerization inhibitors with potent antitumor activities. *European journal of medicinal chemistry* **2021,** 216, 113316.
13. Wang, C.; Gao, M.; Liu, S.; Zou, Z.; Ren, R.; Zhang, C.; Xie, H.; Sun, J.; Qi, Y.; Qu, Q.; Song, Z.; Yang, G.; Wang, H., Pyxinol bearing amino acid residues: Easily achievable and promising modulators of P-glycoprotein-mediated multidrug resistance. *European journal of medicinal chemistry* **2021,** 216, 113317.
14. Lee, S.; Kim, J.; Jo, J.; Chang, J. W.; Sim, J.; Yun, H., Recent advances in development of hetero-bivalent kinase inhibitors. *European journal of medicinal chemistry* **2021,** 216, 113318.
15. Li, Z.; Xin, W.; Wang, Q.; Zhu, M.; Zhou, H., Design and synthesis of N-(3-sulfamoylphenyl)amides as Trypanosoma brucei leucyl-tRNA synthetase inhibitors. *European journal of medicinal chemistry* **2021,** 217, 113319.
16. Srivastava, S.; Ahmad, R.; Khare, S. K., Alzheimer's disease and its treatment by different approaches: A review. *European journal of medicinal chemistry* **2021,** 216, 113320.
17. Aaghaz, S.; Sharma, K.; Jain, R.; Kamal, A., beta-Carbolines as potential anticancer agents. *European journal of medicinal chemistry* **2021,** 216, 113321.

18. Li, Y. S.; He, M.; Zhou, T. S.; Wang, Q.; He, L.; Wang, S. J.; Hu, B.; Wei, B.; Wang, H.; Cui, Z. N., 2,5-Disubstituted furan derivatives containing 1,3,4-thiadiazole moiety as potent alpha-glucosidase and E. coli beta-glucuronidase inhibitors. *European journal of medicinal chemistry* **2021,** 216, 113322.
19. Jo, J.; Lee, D.; Park, Y. H.; Choi, H.; Han, J.; Park, D. H.; Choi, Y. K.; Kwak, J.; Yang, M. K.; Yoo, J. W.; Moon, H. R.; Geum, D.; Kang, K. S.; Yun, H., Discovery and optimization of novel 3-benzyl-N-phenyl-1H-pyrazole-5-carboxamides as bifunctional antidiabetic agents stimulating both insulin secretion and glucose uptake. *European journal of medicinal chemistry* **2021,** 217, 113325.
20. Zhou, W.; Xu, C.; Dong, G.; Qiao, H.; Yang, J.; Liu, H.; Ding, L.; Sun, K.; Zhao, W., Development of phenyltriazole thiol-based derivatives as highly potent inhibitors of DCN1-UBC12 interaction. *European journal of medicinal chemistry* **2021,** 217, 113326.
21. Wu, S.; Xu, C.; Xia, K.; Lin, Y.; Tian, S.; Ma, H.; Ji, Y.; Zhu, F.; He, S.; Zhang, X., Ring closure strategy leads to potent RIPK3 inhibitors. *European journal of medicinal chemistry* **2021,** 217, 113327.
22. Liu, J.; Chen, C.; Wang, D.; Zhang, J.; Zhang, T., Emerging small-molecule inhibitors of the Bruton's tyrosine kinase (BTK): Current development. *European journal of medicinal chemistry* **2021,** 217, 113329.
23. Kayamba, F.; Malimabe, T.; Ademola, I. K.; Pooe, O. J.; Kushwaha, N. D.; Mahlalela, M.; van Zyl, R. L.; Gordon, M.; Mudau, P. T.; Zininga, T.; Shonhai, A.; Nyamori, V. O.; Karpoormath, R., Design and synthesis of quinoline-pyrimidine inspired hybrids as potential plasmodial inhibitors. *European journal of medicinal chemistry* **2021,** 217, 113330.
24. Grieco, I.; Bissaro, M.; Tiz, D. B.; Perez, D. I.; Perez, C.; Martinez, A.; Redenti, S.; Mariotto, E.; Bortolozzi, R.; Viola, G.; Cozza, G.; Spalluto, G.; Moro, S.; Federico, S., Developing novel classes of protein kinase CK1delta inhibitors by fusing [1,2,4]triazole with different bicyclic heteroaromatic systems. *European journal of medicinal chemistry* **2021,** 216, 113331.
25. Gediya, P.; Parikh, P. K.; Vyas, V. K.; Ghate, M. D., Histone deacetylase 2: A potential therapeutic target for cancer and neurodegenerative disorders. *European journal of medicinal chemistry* **2021,** 216, 113332.
26. Gao, D.; Jin, N.; Fu, Y.; Zhu, Y.; Wang, Y.; Wang, T.; Chen, Y.; Zhang, M.; Xiao, Q.; Huang, M.; Li, Y., Rational drug design of benzothiazole-based derivatives as potent signal transducer and activator of transcription 3 (STAT3) signaling pathway inhibitors. *European journal of medicinal chemistry* **2021,** 216, 113333.
27. Dhokne, P.; Sakla, A. P.; Shankaraiah, N., Structural insights of oxindole based kinase inhibitors as anticancer agents: Recent advances. *European journal of medicinal chemistry* **2021,** 216, 113334.
28. Ren, C.; Sun, N.; Kong, Y.; Qu, X.; Liu, H.; Zhong, H.; Song, X.; Yang, X.; Jiang, B., Structure-based discovery of SIAIS001 as an oral bioavailability ALK degrader constructed from Alectinib. *European journal of medicinal chemistry* **2021,** 217, 113335.
29. Kim, G. Y.; Song, C. W.; Yang, Y. S.; Lee, N. R.; Yoo, H. S.; Son, S. H.; Lee, S. J.; Park, J. S.; Lee, J. K.; Inn, K. S.; Kim, N. J., Chemical Degradation of Androgen Receptor (AR) Using Bicalutamide Analog-Thalidomide PROTACs. *Molecules* **2021,** 26, (9).
30. Shim, J. H., Inhibitory Effects of Cycloheterophyllin on Melanin Synthesis. *Molecules* **2021,** 26, (9).
31. Azzouz, Z.; Bettache, A.; Boucherba, N.; Prieto, A.; Martinez, M. J.; Benallaoua, S.; de Eugenio, L. I., Optimization of beta-1,4-Endoxylanase Production by an Aspergillus niger Strain Growing on Wheat Straw and Application in Xylooligosaccharides Production. *Molecules* **2021,** 26, (9).
32. Dipterocarpus. Rubino, F. M., The Redox Potential of the beta-(93)-Cysteine Thiol Group in Human Hemoglobin Estimated from In Vitro Oxidant Challenge Experiments. *Molecules* **2021,** 26, (9).
33. Kim, H.; Yang, W. S.; Htwe, K. M.; Lee, M. N.; Kim, Y. D.; Yoon, K. D.; Lee, B. H.; Lee, S.; Cho, J. Y., Dipterocarpus tuberculatus Roxb. Ethanol Extract Has Anti-Inflammatory and Hepatoprotective Effects In Vitro and In Vivo by Targeting the IRAK1/AP-1 Pathway. *Molecules* **2021,** 26, (9).
34. Budeev, A.; Kantin, G.; Dar'in, D.; Krasavin, M., Diazocarbonyl and Related Compounds in the Synthesis of Azoles. *Molecules* **2021,** 26, (9).
35. Dao, T. B.; Nguyen, T. M.; Nguyen, V. Q.; Tran, T. M.; Tran, N. M.; Nguyen, C. H.; Nguyen, T. H.; Nguyen, H. H.; Sichaem, J.; Tran, C. L.; Duong, T. H., Flavones from Combretum quadrangulare Growing in Vietnam and Their Alpha-Glucosidase Inhibitory Activity. *Molecules* **2021,** 26, (9).

36. Zhen, L.; Lange, H.; Crestini, C., An Analytical Toolbox for Fast and Straightforward Structural Characterisation of Commercially Available Tannins. *Molecules* **2021,** 26, (9).
37. Han, X.; Zhao, L.; Xiang, W.; Qin, C.; Miao, B.; McEachern, D.; Wang, Y.; Metwally, H.; Wang, L.; Matvekas, A.; Wen, B.; Sun, D.; Wang, S., Strategies toward Discovery of Potent and Orally Bioavailable Proteolysis Targeting Chimera Degraders of Androgen Receptor for the Treatment of Prostate Cancer. *Journal of medicinal chemistry* **2021,** 64, (17), 12831-12854.
38. Kato, I.; Ukai, Y.; Kondo, N.; Nozu, K.; Kimura, C.; Hashimoto, K.; Mizusawa, E.; Maki, H.; Naito, A.; Kawai, M., Identification of Thiazoyl Guanidine Derivatives as Novel Antifungal Agents Inhibiting Ergosterol Biosynthesis for Treatment of Invasive Fungal Infections. *Journal of medicinal chemistry* **2021,** 64, (14), 10482-10496.
39. Jimenez, T.; Botero, J.; Otaegui, D.; Calvo, J.; Hernandez, F. J.; San Sebastian, E., Rational Design and Experimental Analysis of Short-Oligonucleotide Substrate Specificity for Targeting Bacterial Nucleases. *Journal of medicinal chemistry* **2021,** 64, (17), 12855-12864.
40. Rossino, G.; Rui, M.; Linciano, P.; Rossi, D.; Boiocchi, M.; Peviani, M.; Poggio, E.; Curti, D.; Schepmann, D.; Wunsch, B.; Gonzalez-Avendano, M.; Vergara-Jaque, A.; Caballero, J.; Collina, S., Bitopic Sigma 1 Receptor Modulators to Shed Light on Molecular Mechanisms Underpinning Ligand Binding and Receptor Oligomerization. *Journal of medicinal chemistry* **2021,** 64, (20), 14997-15016.
41. Plesselova, S.; Garcia-Cerezo, P.; Blanco, V.; Reche-Perez, F. J.; Hernandez-Mateo, F.; Santoyo-Gonzalez, F.; Giron-Gonzalez, M. D.; Salto-Gonzalez, R., Polyethylenimine-Bisphosphonate-Cyclodextrin Ternary Conjugates: Supramolecular Systems for the Delivery of Antineoplastic Drugs. *Journal of medicinal chemistry* **2021,** 64, (16), 12245-12260.
42. Ryan, M. D.; Parkes, A. L.; Corbett, D.; Dickie, A. P.; Southey, M.; Andersen, O. A.; Stein, D. B.; Barbeau, O. R.; Sanzone, A.; Thommes, P.; Barker, J.; Cain, R.; Compper, C.; Dejob, M.; Dorali, A.; Etheridge, D.; Evans, S.; Faulkner, A.; Gadouleau, E.; Gorman, T.; Haase, D.; Holbrow-Wilshaw, M.; Krulle, T.; Li, X.; Lumley, C.; Mertins, B.; Napier, S.; Odedra, R.; Papadopoulos, K.; Roumpelakis, V.; Spear, K.; Trimby, E.; Williams, J.; Zahn, M.; Keefe, A. D.; Zhang, Y.; Soutter, H. T.; Centrella, P. A.; Clark, M. A.; Cuozzo, J. W.; Dumelin, C. E.; Deng, B.; Hunt, A.; Sigel, E. A.; Troast, D. M.; DeJonge, B. L. M., Discovery of Novel UDP-N-Acetylglucosamine Acyltransferase (LpxA) Inhibitors with Activity against Pseudomonas aeruginosa. *Journal of medicinal chemistry* **2021,** 64, (19), 14377-14425.
43. Mann, M. K.; Zepeda-Velazquez, C. A.; Gonzalez-Alvarez, H.; Dong, A.; Kiyota, T.; Aman, A. M.; Loppnau, P.; Li, Y.; Wilson, B.; Arrowsmith, C. H.; Al-Awar, R.; Harding, R. J.; Schapira, M., Structure-Activity Relationship of USP5 Inhibitors. *Journal of medicinal chemistry* **2021,** 64, (20), 15017-15036.
44. Zhang, Z.; Ghosh, A.; Connolly, P. J.; King, P.; Wilde, T.; Wang, J.; Dong, Y.; Li, X.; Liao, D.; Chen, H.; Tian, G.; Suarez, J.; Bonnette, W. G.; Pande, V.; Diloreto, K. A.; Shi, Y.; Patel, S.; Pietrak, B.; Szewczuk, L.; Sensenhauser, C.; Dallas, S.; Edwards, J. P.; Bachman, K. E.; Evans, D. C., Gut-Restricted Selective Cyclooxygenase-2 (COX-2) Inhibitors for Chemoprevention of Colorectal Cancer. *Journal of medicinal chemistry* **2021,** 64, (15), 11570-11596.
45. Singh, H.; Thirupathi, A.; Das, B.; Janni, M.; Kumari, R.; Singh, S.; Rashid, M.; Wahajuddin, M.; Balamurali, M. M.; Jagavelu, K.; Peruncheralathan, S., 2,3-Difunctionalized Benzo[b]thiophene Scaffolds Possessing Potent Antiangiogenic Properties. *Journal of medicinal chemistry* **2022,** 65, (1), 120-134.
46. Dong, G.; Ding, Y.; He, S.; Sheng, C., Molecular Glues for Targeted Protein Degradation: From Serendipity to Rational Discovery. *Journal of medicinal chemistry* **2021,** 64, (15), 10606-10620.
47. Facchini, F. A.; Minotti, A.; Luraghi, A.; Romerio, A.; Gotri, N.; Matamoros-Recio, A.; Iannucci, A.; Palmer, C.; Wang, G.; Ingram, R.; Martin-Santamaria, S.; Pirianov, G.; De Andrea, M.; Valvano, M. A.; Peri, F., Synthetic Glycolipids as Molecular Vaccine Adjuvants: Mechanism of Action in Human Cells and In Vivo Activity. *Journal of medicinal chemistry* **2021,** 64, (16), 12261-12272.
48. Xiang, W.; Zhao, L.; Han, X.; Qin, C.; Miao, B.; McEachern, D.; Wang, Y.; Metwally, H.; Kirchhoff, P. D.; Wang, L.; Matvekas, A.; He, M.; Wen, B.; Sun, D.; Wang, S., Discovery of ARD-2585 as an Exceptionally Potent and Orally Active PROTAC Degrader of Androgen Receptor for the Treatment of Advanced Prostate Cancer. *Journal of medicinal chemistry* **2021,** 64, (18), 13487-13509.

49. Chen, H.; Liu, J.; Kaniskan, H. U.; Wei, W.; Jin, J., Folate-Guided Protein Degradation by Immunomodulatory Imide Drug-Based Molecular Glues and Proteolysis Targeting Chimeras. *Journal of medicinal chemistry* **2021,** 64, (16), 12273-12285.
50. Bowden, G. D.; Stotz, S.; Kinzler, J.; Geibel, C.; Lammerhofer, M.; Pichler, B. J.; Maurer, A., DoE Optimization Empowers the Automated Preparation of Enantiomerically Pure [(18)F]Talazoparib and its In Vivo Evaluation as a PARP Radiotracer. *Journal of medicinal chemistry* **2021,** 64, (21), 15690-15701.
51. Bursavich, M. G.; Harrison, B. A.; Acharya, R.; Costa, D. E.; Freeman, E. A.; Hrdlicka, L. A.; Jin, H.; Kapadnis, S.; Moffit, J. S.; Murphy, D.; Nolan, S. J.; Patzke, H.; Tang, C.; Van Voorhies, H. E.; Wen, M.; Koenig, G.; Blain, J. F.; Burnett, D. A., Discovery of the Oxadiazine FRM-024: A Potent CNS-Penetrant Gamma Secretase Modulator. *Journal of medicinal chemistry* **2021,** 64, (19), 14426-14447.
52. Heightman, T. D.; Berdini, V.; Bevan, L.; Buck, I. M.; Carr, M. G.; Courtin, A.; Coyle, J. E.; Day, J. E. H.; East, C.; Fazal, L.; Griffiths-Jones, C. M.; Howard, S.; Kucia-Tran, J.; Martins, V.; Muench, S.; Munck, J. M.; Norton, D.; O'Reilly, M.; Palmer, N.; Pathuri, P.; Peakman, T. M.; Reader, M.; Rees, D. C.; Rich, S. J.; Shah, A.; Wallis, N. G.; Walton, H.; Wilsher, N. E.; Woolford, A. J.; Cooke, M.; Cousin, D.; Onions, S.; Shannon, J.; Watts, J.; Murray, C. W., Discovery of ASTX029, A Clinical Candidate Which Modulates the Phosphorylation and Catalytic Activity of ERK1/2. *Journal of medicinal chemistry* **2021,** 64, (16), 12286-12303.
53. Xiong, B.; Wang, Y.; Chen, Y.; Xing, S.; Liao, Q.; Chen, Y.; Li, Q.; Li, W.; Sun, H., Strategies for Structural Modification of Small Molecules to Improve Blood-Brain Barrier Penetration: A Recent Perspective. *Journal of medicinal chemistry* **2021,** 64, (18), 13152-13173.
54. Deng, X.; Salgado-Polo, F.; Shao, T.; Xiao, Z.; Van, R.; Chen, J.; Rong, J.; Haider, A.; Shao, Y.; Josephson, L.; Perrakis, A.; Liang, S. H., Imaging Autotaxin In Vivo with (18)F-Labeled Positron Emission Tomography Ligands. *Journal of medicinal chemistry* **2021,** 64, (20), 15053-15068.
55. Fallica, A. N.; Barbaraci, C.; Amata, E.; Pasquinucci, L.; Turnaturi, R.; Dichiara, M.; Intagliata, S.; Gariboldi, M. B.; Marras, E.; Orlandi, V. T.; Ferroni, C.; Martini, C.; Rescifina, A.; Gentile, D.; Varchi, G.; Marrazzo, A., Nitric Oxide Photo-Donor Hybrids of Ciprofloxacin and Norfloxacin: A Shift in Activity from Antimicrobial to Anticancer Agents. *Journal of medicinal chemistry* **2021,** 64, (15), 11597-11613.
56. Monsarrat, C.; Compain, G.; Andre, C.; Engilberge, S.; Martiel, I.; Olieric, V.; Wolff, P.; Brillet, K.; Landolfo, M.; Silva da Veiga, C.; Wagner, J.; Guichard, G.; Burnouf, D. Y., Iterative Structure-Based Optimization of Short Peptides Targeting the Bacterial Sliding Clamp. *Journal of medicinal chemistry* **2021,** 64, (23), 17063-17078.
57. McCoull, W.; Boyd, S.; Brown, M. R.; Coen, M.; Collingwood, O.; Davies, N. L.; Doherty, A.; Fairley, G.; Goldberg, K.; Hardaker, E.; He, G.; Hennessy, E. J.; Hopcroft, P.; Hodgson, G.; Jackson, A.; Jiang, X.; Karmokar, A.; Laine, A. L.; Lindsay, N.; Mao, Y.; Markandu, R.; McMurray, L.; McLean, N.; Mooney, L.; Musgrove, H.; Nissink, J. W. M.; Pflug, A.; Reddy, V. P.; Rawlins, P. B.; Rivers, E.; Schimpl, M.; Smith, G. F.; Tentarelli, S.; Travers, J.; Troup, R. I.; Walton, J.; Wang, C.; Wilkinson, S.; Williamson, B.; Winter-Holt, J.; Yang, D.; Zheng, Y.; Zhu, Q.; Smith, P. D., Optimization of an Imidazo[1,2-a]pyridine Series to Afford Highly Selective Type I1/2 Dual Mer/Axl Kinase Inhibitors with In Vivo Efficacy. *Journal of medicinal chemistry* **2021,** 64, (18), 13524-13539.
58. Liang, J. J.; Xie, H.; Yang, R. H.; Wang, N.; Zheng, Z. J.; Zhou, C.; Wang, Y. L.; Wang, Z. J.; Liu, H. M.; Shan, L. H.; Ke, Y., Designed, synthesized and biological evaluation of proteolysis targeting chimeras (PROTACs) as AR degraders for prostate cancer treatment. *Bioorganic & medicinal chemistry* **2021,** 45, 116331.
59. Cristina Mendonca Nogueira, T.; Vinicius Nora de Souza, M., New FDA oncology small molecule drugs approvals in 2020: Mechanism of action and clinical applications. *Bioorganic & medicinal chemistry* **2021,** 46, 116340.
60. Sun, J.; Wang, J.; Wang, X.; Hu, X.; Cao, H.; Bai, J.; Li, D.; Hua, H., Design and synthesis of beta-carboline derivatives with nitrogen mustard moieties against breast cancer. *Bioorganic & medicinal chemistry* **2021,** 45, 116341.
61. Jiang, Y.; Liu, S.; Tian, G.; Cheung, H. J. H.; Li, X.; Li, X. D., Concise solid-phase synthesis enables derivatisation of YEATS domain cyclopeptide inhibitors for improved cellular uptake. *Bioorganic & medicinal chemistry* **2021,** 45, 116342.

62. Sato, J.; Kusano, H.; Aoki, T.; Shibuya, S.; Yokoo, K.; Komano, K.; Oguma, T.; Matsumoto, S.; Sato, T.; Yasuo, K.; Yamawaki, K., A novel tricyclic beta-lactam exhibiting potent antibacterial activities against carbapenem-resistant Enterobacterales: Synthesis and structure-activity-relationships. *Bioorganic & medicinal chemistry* **2021,** 46, 116343.
63. Yao, H.; Guo, Q.; Wang, M.; Wang, R.; Xu, Z., Discovery of pyrazole N-aryl sulfonate: A novel and highly potent cyclooxygenase-2 (COX-2) selective inhibitors. *Bioorganic & medicinal chemistry* **2021,** 46, 116344.
64. Chia, J. Y.; Miki, T.; Mihara, H.; Tsutsumi, H., Biofunctional supramolecular hydrogels fabricated from a short self-assembling peptide modified with bioactive sequences for the 3D culture of breast cancer MCF-7 cells. *Bioorganic & medicinal chemistry* **2021,** 46, 116345.
65. Yang, H.; Li, Q.; Su, M.; Luo, F.; Liu, Y.; Wang, D.; Fan, Y., Design, synthesis, and biological evaluation of novel 6-(pyridin-3-yl) quinazolin-4(3H)-one derivatives as potential anticancer agents via PI3K inhibition. *Bioorganic & medicinal chemistry* **2021,** 46, 116346.
66. Tsai, W. C.; Aleem, A. M.; Tena, J.; Rivera-Velazquez, M.; Brah, H. S.; Tripathi, S.; D'Silva, M.; Nadler, J. L.; Kalyanaraman, C.; Jacobson, M. P.; Holman, T., Docking and mutagenesis studies lead to improved inhibitor development of ML355 for human platelet 12-lipoxygenase. *Bioorganic & medicinal chemistry* **2021,** 46, 116347.
67. Toviwek, B.; Phuangsawai, O.; Konsue, A.; Hannongbua, S.; Riley, J.; Mutter, N.; Anderson, M.; Webster, L.; Hallyburton, I.; Read, K. D.; Gleeson, M. P., Preparation, biological & cheminformatics-based assessment of N(2),N(4)-diphenylpyrimidine-2,4-diamine as potential Kinase-targeted antimalarials. *Bioorganic & medicinal chemistry* **2021,** 46, 116348.
68. Tsai, W. C.; Gilbert, N. C.; Ohler, A.; Armstrong, M.; Perry, S.; Kalyanaraman, C.; Yasgar, A.; Rai, G.; Simeonov, A.; Jadhav, A.; Standley, M.; Lee, H. W.; Crews, P.; Iavarone, A. T.; Jacobson, M. P.; Neau, D. B.; Offenbacher, A. R.; Newcomer, M.; Holman, T. R., Kinetic and structural investigations of novel inhibitors of human epithelial 15-lipoxygenase-2. *Bioorganic & medicinal chemistry* **2021,** 46, 116349.
69. Wang, B.; Feng, W.; Wang, J.; Dong, Y.; Liu, Y.; Yao, Y.; Zhang, J.; Shi, W.; Liu, L.; Zhang, H.; He, X.; Chang, X.; Wang, X.; Xu, H.; Liu, F.; Feng, J., Discovery of potent and selective Bcl-2 inhibitors with acyl sulfonamide skeleton. *Bioorganic & medicinal chemistry* **2021,** 47, 116350.
70. Biteau, N. G.; Roy, V.; Lambry, J. C.; Becker, H. F.; Myllykallio, H.; Agrofoglio, L. A., Synthesis of acyclic nucleoside phosphonates targeting flavin-dependent thymidylate synthase in Mycobacterium tuberculosis. *Bioorganic & medicinal chemistry* **2021,** 46, 116351.
71. Ma, Z.; Jiang, L.; Li, B.; Liang, D.; Feng, Y.; Liu, L.; Jiang, C., Discovery of benzimidazole derivatives as potent and selective aldehyde dehydrogenase 1A1 (ALDH1A1) inhibitors with glucose consumption improving activity. *Bioorganic & medicinal chemistry* **2021,** 46, 116352.
72. Chang, T. C.; Tanaka, K., In vivo organic synthesis by metal catalysts. *Bioorganic & medicinal chemistry* **2021,** 46, 116353.
73. Kumar, S.; Mittal, A.; Mittal, A., A review upon medicinal perspective and designing rationale of DPP-4 inhibitors. *Bioorganic & medicinal chemistry* **2021,** 46, 116354.
74. Schwarthoff, S.; Tischer, N.; Sager, H.; Schatz, B.; Rohrbach, M. M.; Raztsou, I.; Robaa, D.; Gaube, F.; Arndt, H. D.; Winckler, T., Evaluation of gamma-carboline-phenothiazine conjugates as simultaneous NMDA receptor blockers and cholinesterase inhibitors. *Bioorganic & medicinal chemistry* **2021,** 46, 116355.
75. Mahajan, S.; Choudhary, S.; Kumar, P.; Tomar, S., Antiviral strategies targeting host factors and mechanisms obliging +ssRNA viral pathogens. *Bioorganic & medicinal chemistry* **2021,** 46, 116356.
76. Yudi Utomo, R.; Asawa, Y.; Okada, S.; Ban, H. S.; Yoshimori, A.; Bajorath, J.; Nakamura, H., Development of curcumin-based amyloid beta aggregation inhibitors for Alzheimer's disease using the SAR matrix approach. *Bioorganic & medicinal chemistry* **2021,** 46, 116357.
77. Liu, X. J.; Xu, L.; Pang, X. J.; Ying Yuan, X.; Yu, G. X.; Li, Y. R.; Guan, Y. F.; Zhang, Y. B.; Song, J.; Zhang, Q. R.; Zhang, S. Y., Progress in the development of small molecular inhibitors of the Bruton's tyrosine kinase (BTK) as a promising cancer therapy. *Bioorganic & medicinal chemistry* **2021,** 47, 116358.
78. Hopkins, B. T.; Bame, E.; Bajrami, B.; Black, C.; Bohnert, T.; Boiselle, C.; Burdette, D.; Burns, J. C.; Delva, L.; Donaldson, D.; Grater, R.; Gu, C.; Hoemberger, M.; Johnson, J.; Kapadnis, S.; King, K.; Lulla, M.; Ma, B.; Marx, I.; Magee, T.; Meissner, R.; Metrick, C. M.; Mingueneau, M.; Murugan, P.; Otipoby, K. L.; Polack, E.; Poreci, U.; Prince, R.; Roach, A. M.; Rowbottom, C.; Santoro, J. C.; Schroeder, P.; Tang, H.; Tien, E.; Zhang, F.;

Lyssikatos, J., Discovery and Preclinical Characterization of BIIB091, a Reversible, Selective BTK Inhibitor for the Treatment of Multiple Sclerosis. *Journal of medicinal chemistry* **2022,** 65, (2), 1206-1224.

79. Tong, X.; Liu, X.; Tan, X.; Li, X.; Jiang, J.; Xiong, Z.; Xu, T.; Jiang, H.; Qiao, N.; Zheng, M., Generative Models for De Novo Drug Design. *Journal of medicinal chemistry* **2021,** 64, (19), 14011-14027.
80. Zheng, L.; Ren, R.; Sun, X.; Zou, Y.; Shi, Y.; Di, B.; Niu, M. M., Discovery of a Dual Tubulin and Poly(ADP-Ribose) Polymerase-1 Inhibitor by Structure-Based Pharmacophore Modeling, Virtual Screening, Molecular Docking, and Biological Evaluation. *Journal of medicinal chemistry* **2021,** 64, (21), 15702-15715.
81. Cui, H.; Carlson, A. S.; Schleiff, M. A.; Divakaran, A.; Johnson, J. A.; Buchholz, C. R.; Zahid, H.; Vail, N. R.; Shi, K.; Aihara, H.; Harki, D. A.; Miller, G. P.; Topczewski, J. J.; Pomerantz, W. C. K., 4-Methyl-1,2,3-Triazoles as N-Acetyl-Lysine Mimics Afford Potent BET Bromodomain Inhibitors with Improved Selectivity. *Journal of medicinal chemistry* **2021,** 64, (14), 10497-10511.
82. Munoz, E.; Chen, G.; Hossain, A.; Wu, S.; Oceguera Nava, E.; Hang, J.; Lee, T.; Zhang, Q.; Wang, G.; Chen, Q. H., Synthesis and biological evaluation of niclosamide PROTACs. *Bioorganic & medicinal chemistry letters* **2022,** 72, 128870.
83. Luo, W.; Huang, Z.; Xu, D.; Yang, M.; Zhu, Y.; Shen, L.; Chen, S.; Tao, X.; Bin, W.; Hu, Y.; Franzblau, S. G.; Jiang, N.; Wei, Y.; Wei, X.; Ding, C. Z., Discovery and preclinical evaluations of JBD0131, a novel nitrodihydro-imidazooxazole anti-tuberculosis agent. *Bioorganic & medicinal chemistry letters* **2022,** 72, 128871.
84. Gockel, L. M.; Pfeifer, V.; Baltes, F.; Bachmaier, R. D.; Wagner, K. G.; Bendas, G.; Gutschow, M.; Sosic, I.; Steinebach, C., Design, synthesis, and characterization of PROTACs targeting the androgen receptor in prostate and lung cancer models. *Archiv der Pharmazie* **2022,** 355, (5), e2100467.
85. Mykhailenko, O.; Bezruk, I.; Ivanauskas, L.; Georgiyants, V., Comparative analysis of apocarotenoids and phenolic constituents of Crocus sativus stigmas from 11 countries: Ecological impact. *Archiv der Pharmazie* **2022,** 355, (4), e2100468.
86. Wang, R., Current perspectives on naturally occurring saponins as anticancer agents. *Archiv der Pharmazie* **2022,** 355, (5), e2100469.
87. Maher, M.; Zaher, A. F.; Mahmoud, Z.; Kassab, A. E., Recent green approaches for the synthesis of pyrazolo[3,4-d]pyrimidines: A mini review. *Archiv der Pharmazie* **2022,** 355, (6), e2100470.
88. Aggul, A. G.; Uzun, N.; Kuzu, M.; Taslimi, P.; Gulcin, I., Some phenolic natural compounds as carbonic anhydrase inhibitors: An in vitro and in silico study. *Archiv der Pharmazie* **2022,** 355, (6), e2100476.
89. Solangi, M.; Kanwal; Khan, K. M.; Chigurupati, S.; Saleem, F.; Qureshi, U.; Ul-Haq, Z.; Jabeen, A.; Felemban, S. G.; Zafar, F.; Perveen, S.; Taha, M.; Bhatia, S., Isatin thiazoles as antidiabetic: Synthesis, in vitro enzyme inhibitory activities, kinetics, and in silico studies. *Archiv der Pharmazie* **2022,** 355, (6), e2100481.
